# The structural and functional brain alternations in tobacco use disorder: a systematic review and meta-analysis

**DOI:** 10.3389/fpsyt.2025.1403604

**Published:** 2025-04-11

**Authors:** Longyao Ma, Qiuying Tao, Jinghan Dang, Jieping Sun, Xiaoyu Niu, Mengzhe Zhang, Yimeng Kang, Weijian Wang, Jingliang Cheng, Yong Zhang

**Affiliations:** ^1^ Department of Magnetic Resonance Imaging, The First Affiliated Hospital of Zhengzhou University, Zhengzhou, China; ^2^ Zhengzhou Key Laboratory of Brain Function and Cognitive Magnetic Resonance Imaging, Zhengzhou, China

**Keywords:** meta-analysis, tobacco use disorder, voxel-based morphometry, functional magnetic resonance imaging, multimodel neuroimaging

## Abstract

**Background:**

While numerous previous studies have indicated that nicotine intake results in gray matter and functional brain abnormalities in tobacco use disorder (TUD), the majority of results could not be replicated or even reversed. Consequently, it is important to utilize relevant coordinate data for a comprehensive meta-analysis to identify the shared patterns of structural, functional, and multimodal alternations in TUD.

**Method:**

The present study conducted a systematic retrieval of studies published on PubMed, Web of Science, and Scopus from January 1, 2010, to December 12, 2023, to identify studies on voxel-based morphometry (VBM) and resting-state functional magnetic resonance imaging (rs-fMRI) for TUD. Then, two meta-analyses using the anisotropic seed-based d mapping method were used to detect brain comprehensive alterations in individuals with TUD. Furthermore, two meta-analyses were pooled for multimodal analysis to discover multimodal anomalies. Finally, subgroup analyses were performed to explore the sources of TUD heterogeneity from both methodological and age perspectives.

**Result:**

This study encompassed a total of 25 VBM studies, including 1,249 individuals with TUD and 1,874 healthy controls (HCs), and 35 rs-fMRI studies, including 1,436 individuals with TUD and 1,550 HCs. For rs-fMRI analysis, individuals with TUD exhibited increased intrinsic function in the right cerebellum crus2, left superior frontal gyrus, left inferior parietal gyrus, and left supplementary motor area and decreased intrinsic function in the right gyrus rectus, right superior/middle frontal gyrus, and left inferior frontal gyrus. For VBM analysis, individuals with TUD showed decreased gray matter volume (GMV) in the left superior temporal gyrus, right superior frontal gyrus, right anterior cingulate/paracingulate gyrus, left superior frontal gyrus, and right anterior thalamic region and increased GMV in the right lingual gyrus.

**Conclusion:**

This meta-analysis illustrates structural and functional abnormalities of the default mode network, executive control network, and salience network in individuals with TUD. Multimodal analysis of the right lingual gyrus provided additional information, offering the potential for identifying more therapeutic targets for interventions against TUD.

## Introduction

Tobacco is currently acknowledged as a global public health issue, with its significant impact on people’s health being widely recognized. Smokers have a mortality rate three times higher than non-smokers, with approximately 60% of the deaths attributed to smoking-related diseases (1). Individuals with tobacco use disorder (TUD) typically exhibit overwhelming motivational intensity and decreased capacity to regulate their craving for tobacco (2). As a result, this imposes a considerable economic burden on individuals, society, and the nation.

In 2003, the World Health Organization (WHO) adopted the Tobacco Convention (3). Studies have shown that smoking is linked to cognitive impairments and decline, which raises the risk of developing late-life dementia (4, 5). Moreover, tobacco has been found to decrease fertility, increase the risk of miscarriage, and even lead to asthma and cancer, significantly impacting the development of unborn infants (6). This phenomenon could potentially be attributed to the presence of more than 4,000 chemical components and additives found in tobacco (6). Despite the multitude of hazards associated with tobacco, the rate of cessation remains alarmingly low at a mere 3.5% (7). A significant number of individuals face challenges in their attempts to quit smoking, often resulting in quick relapses back into smoking habits (8, 9). Therefore, the investigation of the mechanisms that underlie the occurrence and development of TUD holds significant implications.

Over the past decade, significant advancements in magnetic resonance technology have enabled numerous researchers to uncover structural and functional disparities in the brains of individuals with TUD (10–12). Presently, analytical methods in neuroimaging can be mainly split into two categories: the first one is used to describe abnormalities in brain structural imaging (gray matter or white matter), such as voxel/surface-based morphological (VBM/SBM) analysis and tract-based spatial statistics (TBSS) (13, 14). The second is for describing abnormalities in functional imaging between remote and regional brain activities, such as seed-based functional connectivity (FC), amplitude of low-frequency fluctuation (ALFF), fractional ALFF, and regional homogeneity (ReHo) (15–18). Structurally, the majority of studies have employed VBM to measure gray matter volume (GMV) differences to investigate the pathophysiological mechanisms of TUD (19, 20). Over time, GMV can function not only as a relatively stable indicator but also as a foundation for alternations in neural activity within the brain. Compared to healthy controls, the study has revealed that individuals with TUD exhibit increased GMV in the right lingual gyrus and left occipital cortex/cuneus (21). Functionally, ALFF, fractional ALFF (fALFF), ReHo, and FC, as the most common indicators of spontaneous alternations in brain activity, have been widely used in addictive disorders to explore the underlying neurobiological mechanisms and have proven to be valuable tools in the study of TUD-related neural alterations (22–24). However, the results of the functional magnetic resonance imaging (fMRI) studies showed more significant functional alternations in the sensorimotor area, specifically in the anterior and posterior cingulate gyrus, in individuals with TUD compared to healthy controls (25–28). This could be due to various factors, including differences in study design, sample characteristics, data analysis methods, and variations in the severity and duration of TUD among participants. Although there have been neuroimaging meta-analyses that have explored structural or functional differences in individuals with TUD, the following questions remain in the meta-analysis on TUD: first, a large number of innovative and high-quality articles have appeared on this topic, so it is time to rerun the meta-analysis to complement or revise the previous results. Second, previous meta-analyses have analyzed structure or function separately and have not synthesized their findings for multimodal analysis to discover more information. Finally, several meta-analyses were missing or insufficient for subgroup analyses and sources of heterogeneity, so we will develop our study in terms of methodology and age. Additionally, TUD is a complex disorder influenced by multiple interacting factors, including genetic, environmental, and behavioral factors. This complexity makes it challenging to pinpoint specific brain regions and their exact roles in TUD (29).

Therefore, to address these problems, the main objectives of our study are as follows: 1) collecting larger sample sizes, employing standardized methodologies, and replicating studies; 2) two meta-analyses of all collected VBM and resting-state fMRI (rs-fMRI) studies were performed using the seed-based d mapping (SDM) package, and further multimodal analyses were performed using p-value images from the aforementioned meta-analyses. The integration of findings may contribute to a more comprehensive understanding of the neural mechanisms behind TUD. 3) Separate subgroup analyses of rs-fMRI study methods and age (adults and adolescents) were performed for all studies. This approach allows us to examine the influence of these variables and assess their impact on the observed heterogeneity in the data. Therefore, our study is the most current and the most comprehensive. Based on previous studies, we hypothesized that the lingual gyrus in individuals with TUD would develop disorder-specific GMV abnormalities. As for rs-fMRI, we hypothesized that individuals with TUD exhibit abnormal under-activation of the anterior and posterior cingulate gyrus.

## Method

### Literature search and selection criteria

Systematic and comprehensive searches were conducted in PubMed, Web of Science, and Scopus databases from January 1, 2010, to December 12, 2023, using several keywords (“smoking” or “nicotine” or “tobacco” or “cigarette” or “smokers”) and (“rs-fMRI” or “resting-state functional magnetic resonance imaging”) or (“ALFF” or “amplitude of low-frequency fluctuations”) or (“ReHo” or “regional homogeneity”) or (“fALFF” or “fractional amplitude of low-frequency fluctuations”) or (“FC” or “functional connectivity”) or (“VBM” or “voxel-based morphometry” or “gray matter”).

### Study selection

The study was included according to the following criteria. 1) It was an original article published in English in a peer-reviewed journal. 2) The individual with TUD was without other diseases such as hypertension, diabetes, cerebrovascular, and multiple sclerosis. 3) Task-free acquisition of scans were acquired. 4) The analysis was carried out with rs-fMRI and VBM between individuals with TUD and HCs. 5) The whole-brain results were reported using stereotactic three-dimensional coordinates (x, y, z), defined by either Talairach or the Montreal Neurological Institute. 6) A significance threshold was used. Meanwhile, studies that met one of the following criteria were excluded: 1) meta-analysis or reviews, 2) peak coordinates not reported, 3) no control group, 4) less than 10 subjects, 5) non-empirical or non-human, and 6) did not use rs-fMRI or VBM.

### Data extraction and assessment

The current meta-analysis is based on the guideline of Preferred Reporting Items for Systematic Reviews and Meta-Analyses (PRISMA). Three authors (Longyao Ma, Qiuying Tao, and Jinghan Dang) independently selected the peak coordinates and effect sizes of the difference in brain (measured by rs-fMRI and VBM) between individuals with TUD and HCs by inspecting the abstracts and evaluating the quality of articles. Any differences were resolved through a joint reassessment of the research study with a corresponding author (Yong Zhang). In addition, the relevant sample features (sample size, gender, age, and education levels) and clinical characteristics (smoking years, cigarettes/day, pack-years, and Fagerstrom Test for Nicotine Dependence (FTND)) were extracted. Technical details (MRI scanner, software, scanner parameters, and threshold for correction) were also recorded. In total, 35 rs-fMRI studies (37 datasets) and 25 VBM studies (28 datasets) were included in this meta-analysis.

### Statistical analysis

The differences in rs-fMRI and VBM between individuals with TUD and HCs were analyzed by the anisotropic effect-size version of the SDM software package (version 5.15), which is freely available for use at the following URL (http://www.sdmproject.com/software). SDM is a meta-analysis method that aims to combine the reported peak coordinates from various studies to recreate an effect size map comparing individuals with TUD to HCs (30).

According to the SDM tutorial and standard steps, voxel meta-analysis in the SDM software is initiated gradually (http://www.sdmproject.com/software/tutorial.pdf). The steps were as follows: 1) a text file including reported peak coordinates and effect sizes (t-values or z-scores) of differences in rs-fMRI and VBM between individuals with TUD and HCs was prepared. 2) p-Values or z-scores in certain research studies needed to be converted into t-values online (http://www.sdmproject.com/utilities/?show=Statistics). If there was no effect size, “p” for positive peaks and “n” for negative peaks were used instead. 3) Peak coordinates were converted into standardized Montreal Neurological Institute (MNI) space. 4) An anisotropic unnormalized Gaussian kernel was used with a 20-mm full width at half maximum, a random-effect linear model, and the following default thresholds: voxel p < 0.005, peak height threshold > 1, and cluster extent threshold > 10 voxels for balancing sensitivity and specificity (30, 31). 5) The whole-brain jackknife sensitivity analysis was performed to verify the stability, reliability, and significance of the results. 6) The Q statistic was utilized to examine inter-study variation in the results obtained from the meta-analysis and assess heterogeneity (p = 0.005, peak z = 1, cluster extent = 10 voxels). 7) Egger’s test was used to assess the possibility of publication bias and further evaluate the degree of asymmetry present in the funnel plot (32) (**Supplementary Figure S1**).

In this study, two analyses of VBM and rs-fMRI were performed to ascertain patterns of routine and specific brain neurological alternations in individuals with TUD. Subsequently, a multimodal analysis was carried out using p-value images from the two analyses with the aim of identifying overlapping regions of structural and functional brain abnormalities in individuals with TUD.

### Subgroup analysis for age and methods

In order to enhance the reliability and stability of the findings and investigate potential sources of heterogeneity, subgroup analyses were performed for studies focusing on ALFF, fALFF, ReHo, and FC in rs-fMRI. The subgroups identified in the meta-analysis can be further employed to explore potential confounding factors and identify anomalies in brain function among individuals with TUD. This approach enables a more detailed investigation of the data, allowing for the identification of any underlying factors that may contribute to the observed effects.

In addition, to obtain more about the source of heterogeneity from an age perspective, we also performed subgroup analyses by dividing the study subjects into two distinct subtypes: the adult group (>24 years old) and the adolescent group (<24 years old) (33).

## Result

### Included studies and sample characteristics

**Figure 1:**
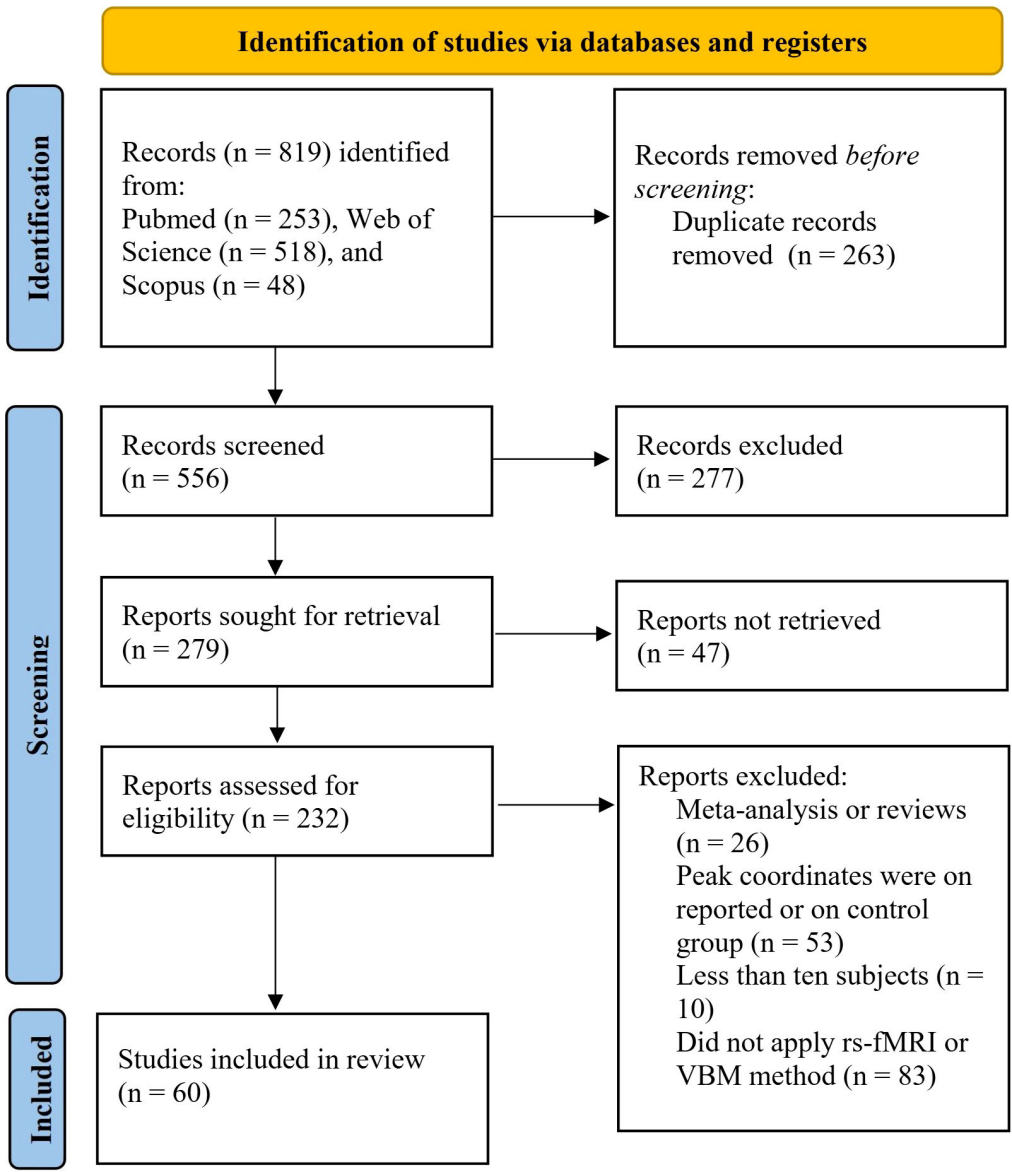
Flowchart of study selection for the meta-analyses 25 VBM studies (28 datasets) and 35 rs-fMRI studies (37 datasets) included for meta-analysis. VBM, voxel-based morphometry; rs-fMRI, resting-state functional magnetic resonance imaging.

The flowchart of this study is shown in **Figure 1**. According to the inclusion criteria, this meta-analysis included a total of 60 studies (65 datasets), which comprised 25 VBM studies (28 datasets), including 1,249 individuals with TUD and 1,874 HCs, and 35 rs-fMRI studies (37 datasets), including 1,436 individuals with TUD and 1550 HCs. There are 20 studies using the resting-state FC method (907 individuals with TUD and 979 HCs) and 15 studies using the ReHo, ALFF, and fALFF methods (529 individuals with TUD and 571 HCs). More samples’ demographic and clinical characteristics are shown in **Table 1** and **Supplementary Table S1**.

**Table 1:** Demographic characteristics of rs-fMRI and VBM studies in individuals with TUD.

Study	Individuals with TUD			HCs			Method	Diagnostic
	Sample size (female)	Mean age (years)	Mean education levels (years)	Sample size (female)	Mean age (years)	Mean education levels (years)		
(1) rs-fMRI studies								
Akkermans et al. (25)	25 (7)	22.56 (2.84)	NA	23 (9)	21.74 (1.82)	NA	Seed-based FC	DSM-IV
Bi et al. (22)	40 (NA)	19.62 (1.89)	12.05 (1.32)	40 (NA)	19.80 (2.04)	12.25 (1.52)	Seed-based FC	DSM-5
Chen et al. (34)	29 (NA)	22.14 (2.54)	10.17 (1.91)	22 (NA)	21.00 (2.33)	11.00 (1.37)	Seed-based FC	DSM-IV
Zhou et al. (35)	37 (NA)	33.11 (9.58)	15.08 (3.00)	37 (NA)	32.81 (9.57)	16.64 (1.94)	Seed-based FC	ICD-10
Ge et al. (36)	29 (8)	22.58 (2.41)	13.03 (2.06)	33 (6)	20.78 (2.51)	12.67 (2.58)	Seed-based FC	DSM-IV
Lin et al. (37)	60 (25)	22.54 (2.62)	12.94 (1.96)	67 (28)	22.54 (3.09)	13.82 (3.27)	Seed-based FC	DSM-IV
Niu et al. (38)	86 (NA)	36.03 (7.87)	14.26 (2.46)	56 (NA)	33.96 (7.19)	14.70 (2.51)	Seed-based FC	DSM-5
Qiu et al. (23)	44 (21)	75.83 (7.64)	16.27 (2.61)	30 (18)	72.98 (7.25)	16.88 (2.33)	Seed-based FC	DSM-IV
Qiu et al. (23)	33 (12)	76.61 (7.54)	15.79 (2.40)	130 (56)	74.33 (7.87)	16.71 (2.54)	Seed-based FC	DSM-IV
Shen et al. (24)	85 (NA)	38.24 (6.81)	14.01 (2.94)	41 (NA)	38.46 (8.60)	15.37 (6.58)	Seed-based FC	DSM-IV
Shen et al.(39)	84 (NA)	38.23 (6.58)	15.37 (4.67)	41 (NA)	38.46 (8.60)	15.37 (4.67)	Seed-based FC	DSM-IV
Stoeckel et al. (40)	16 (4)	37.94 (11.61)	14.44 (1.67)	16 (5)	34.19 (7.20)	17.63 (10.49)	Seed-based FC	DSM-IV
Tan et al. (26)	29 (NA)	63.97 (4.75)	13.34 (3.73)	28 (NA)	61.75 (4.98)	14.14 (4.31)	Seed-based FC	DSM-IV
Wang, Bai, et al. (27)	24 (NA)	20.8 (1.80)	12.60 (1.20)	24 (NA)	20.60 (2.50)	12.8 (1.40)	Seed-based FC	DSM-IV
Zhang, Zeng, et al. (41)	29 (17)	76.19 (6.80)	16.24 (1.70)	54 (31)	75.76 (7.48)	16.35 (2.32)	Seed-based FC	DSM-IV
Yip et al. (42)	42 (10)	44.64 (11.20)	NA	60 (34)	29.27 (10.16)	NA	Seed-based FC	DSM-IV
Qiu et al. (43)	44 (21)	75.83 (7.64)	16.27 (2.61)	86 (40)	75.14 (7.85)	16.16 (2.34)	Seed-based FC	DSM-IV
Qiu et al. (43)	32 (12)	76.19 (7.27)	16.03 (1.98)	62 (22)	76.19 (6.83)	16.13 (2.47)	Seed-based FC	DSM-IV
Wang et al. (44)	48 (NA)	19.58 (1.99)	NA	49 (NA)	19.05 (1.72)	NA	ICA-based FC	DSM-5
Claus and Weywadt (45)	35 (18)	56.40 (9.40)	NA	36 (26)	59.80 (8.70)	NA	ICA-based FC	DSM-IV
Zhang et al. (46)	45 (NA)	35.80 (9.90)	14.40 (3.10)	34 (NA)	31.80 (9.60)	15.70 (1.30)	ICA-based FC	DSM-IV
Huang et al. (47)	11 (NA)	23.70 (1.98)	NA	10 (NA)	22.50 (6.78)	NA	ICA-based FC	DSM-IV
Chen and Mo (48)	14 (NA)	34.0 (11.70)	16.60 (2.70)	11 (NA)	34.50 (11.0)	34.50 (11.00)	ReHo	DSM-IV
Yu et al. (17)	16 (NA)	41.6 (5.50)	10.90 (2.20)	16 (NA)	39.20 (4.50)	12.20 (2.50)	ReHo	DSM-IV
Wu et al. (49)	31 (7)	46.29 (7.07)	8.26 (2.25)	33 (5)	46.91 (8.97)	8.79 (2.70)	ReHo	DSM-IV
Tang et al. (50)	45 (8)	27.90 (5.60)	13.10 (3.00)	44 (10)	26.30 (5.80)	15.00 (2.60)	ReHo	DSM-IV
Wang et al. (28)	32 (NA)	38.68 (7.02)	13.81 (2.64)	23 (NA)	40.30 (6.69)	13.34 (2.46)	ReHo	DSM-IV
Wen et al. (51)	56 (24)	22.63 (2.70)	13.0 (2.03)	63 (25)	22.58 (3.12)	13.82 (3.31)	ReHo	DSM-5
Zhang et al. (16)	18 (4)	74.61 (8.84)	15.72 (2.23)	98 (39)	73.67 (6.78)	16.80 (2.60)	ReHo	DSM-IV
Liu et al. (52)	21 (2)	29.05 (10.01)	10.86 (2.44)	21 (6)	30.95 (9.76)	11.05 (3.12)	ALFF	SCID-IP
Qiu et al. (15)	46 (15)	22.88 (2.78)	11.83 (2.50)	60 (19)	22.29 (3.48)	14.76 (3.39)	ALFF	DSM-IV
Niu et al. (38)	86 (NA)	36.03 (7.87)	14.26 (2.46)	56 (NA)	33.96 (7.19)	14.70 (2.51)	ALFF	DSM-5
Gao et al. (53)	30 (NA)	33.03 (7.08)	14.97 (1.77)	24 (NA)	28.50 (4.80)	16.67 (2.14)	ALFF	DSM-IV
Chu et al. (54)	20 (NA)	30.00 (4.00)	17.00 (3.00)	19 (10)	29.00 (5.00)	16.00 (4.00)	fALFF	ICD-10
Wang et al. (18)	55 (NA)	39.40 (6.90)	13.60 (2.60)	49 (NA)	37.30 (8.00)	16.20 (4.50)	fALFF	DSM-IV
Gao et al. (55)	30 (NA)	33.67 (7.18)	15.37 (2.34)	24 (NA)	31.88 (6.99)	16.67 (2.14)	fALFF	DSM-IV
Tan et al.(26)	29 (NA)	63.97 (4.75)	13.34 (3.73)	28 (NA)	61.75 (4.98)	14.14 (4.31)	fALFF	DSM-IV
(2) VBM studies								
Conti (56)	11 (63.6%)	25.2 (9.3)	3.3 (1.0)	24 (45.8%)	28.5 (9.5)	4.2 (0.6)	VBM	DSM-V
Conti (57)	28 (35.7%)	28.1 (8.3)	3.5 (0.7)	24 (45.8%)	28.5 (9.5)	4.2 (0.6)	VBM	DSM-V
Zhang, Gao, et al. (58)	28 (NA)	31.29 (5.56)	15.54 (1.35)	28 (NA)	31.68 (6.57)	16.32 (3.21)	VBM	DSM-V
Daniju et al. (59)	19 (14)	22.8 (3.6)	NA	35 (20)	22.8 (4.9)	NA	VBM	DSM-V
Kunas et al. (60)	62 (34)	31.23 (9.5)	NA	116 (67)	31.85 (10.8)	NA	VBM	DSM-IV
Ye et al. (61)	37 (8)	47.18 (7.22)	9.24 (2.16)	28 (8)	43 (9.62)	11.67 (4.72)	VBM	DSM-IV
Chen et al. (62)	70 (29)	29.79 (3.05)	13.06 (1.87)	209 (80)	29.27 (3.79)	15.11 (1.74)	VBM	DSM-IV
Cai et al. (63)	23 (6)	45.7 (6.8)	9.4 (2.8)	23 (6)	43.8 (9.4)	11.6 (4.5)	VBM	DSM-IV
Bu et al. (64)	26 (0)	21.42 (1.73)	13.92 (0.83)	26 (0)	20.58 (1.47)	13.65 (0.68)	VBM	DSM-V
Franklin et al. (65)	80 (39)	35.7 (11.1)	14.1 (2.0)	80 (39)	33.2 (7.5)	13.6 (2.2)	VBM	DSM-IV
Hanlon et al. (21)	58 (25)	32 (NA)	20.1 (0.3)	60 (27)	29.5 (NA)	20.7 (0.3)	VBM	NA
Fritz et al. (14)	315 (167)	44.10 (11.84)	NA	659 (416)	51.49 (14.45)	NA	VBM	NA
Gallinat et al. (66)	22 (12)	30.8 (7.5)	NA	23 (12)	30.3 (7.9)	NA	VBM	DSM-IV
Morales et al. (67)	25 (13)	35.4 (1.8)	13.6 (0.5)	18 (8)	30.1 (2.2)	13.2 (0.6)	VBM	DSM-IV
Peng et al. (13)	27 (0)	33.26 (3.73)	19.30 (1.32)	53 (0)	30.83 (5.18)	19.33 (1.29)	VBM	ICD-10
Peng et al. (13)	26 (0)	29.42 (4.43)	19.12 (1.48)	53 (0)	30.83 (5.18)	19.33 (1.29)	VBM	ICD-10
Qian et al. (19)	44 (NA)	39 (6.5)	13.7 (2.6)	41 (NA)	38.5 (7.4)	18.9 (6.4)	VBM	DSM-IV
Stoeckel et al. (40)	16 (4)	37.94 (11.61)	14.44 (1.67)	16 (5)	34.19 (7.20)	15.25 (1.00)	VBM	DSM-IV
Wang et al. (68)	22 (0)	22.48 (2.48)	15.14 (1.83)	20 (0)	21.8 (1.32)	15.2 (1.19)	VBM	DSM-IV
Peng et al. (69)	30 (0)	30.7 (4.86)	19.26 (1.63)	53 (0)	30.83 (5.18)	19.33 (1.29)	VBM	ICD-10
Peng et al. (69)	23 (0)	32.74 (4.34)	19.17 (1.80)	53 (0)	30.83 (5.18)	19.33 (1.29)	VBM	ICD-10
Yu (70)	16 (NA)	41.6 (5.5)	10.9 (2.2)	16 (NA)	39.2 (4.5)	12.2 (2.5)	VBM	DSM-IV
Zhang et al. (71)	48 (24)	31.4 (8.1)	13.1 (2.2)	48 (24)	31.1 (8.8)	13.5 (1.8)	VBM	DSM-IV
Zorlu et al. (12)	25 (13)	34.6 (10.1)	9.1 (3.7)	25 (15)	36.7 (10.1)	8.6 (3.7)	VBM	DSM-IV
Shen et al. (39)	85 (0)	38.24 (6.81)	14.01 (2.94)	41 (0)	38.46 (8.60)	15.37 (4.67)	VBM	DSM-IV
Liao et al. (11)	44 (18.2%)	28.1 (5.5)	13.2 (2.92)	44 (22.7%)	26.3 (5.84)	15.0 (2.6)	VBM	DSM-IV
Brody et al. (10)	19 (42.1%)	39.5 (10.3)	NA	17 (41.2%)	37.9 (12.9)	NA	VBM	DSM-IV
Faulkner et al. (20)	12 (8)	25.4 (4.58)	16.69 (2.12)	26 (15)	22.87 (4.60)	15.14 (4.60)	VBM	DSM-IV

TUD, tobacco use disorder; HCs, healthy controls; ReHo, regional homogeneity; ALFF, amplitude of low-frequency fluctuations; fALFF, fractional amplitude of low-frequency fluctuations; FC, functional connectivity; NA, not available; DSM, *Diagnostic and Statistical Manual of Mental Disorders*; rs-fMRI, resting-state functional magnetic resonance imaging; VBM, voxel-based morphometry; ICD-10, The International Statistical Classification of Diseases and Related Health Problems 10th Revision; ICA, independent component analysis; SCID-IP, Structured Clinical Interview for Diagnostic and Statistical Manual of Mental Disorders.

### Meta-analysis results for VBM and rs-fMRI studies

#### VBM meta-analysis in TUD

**Figure 2:**
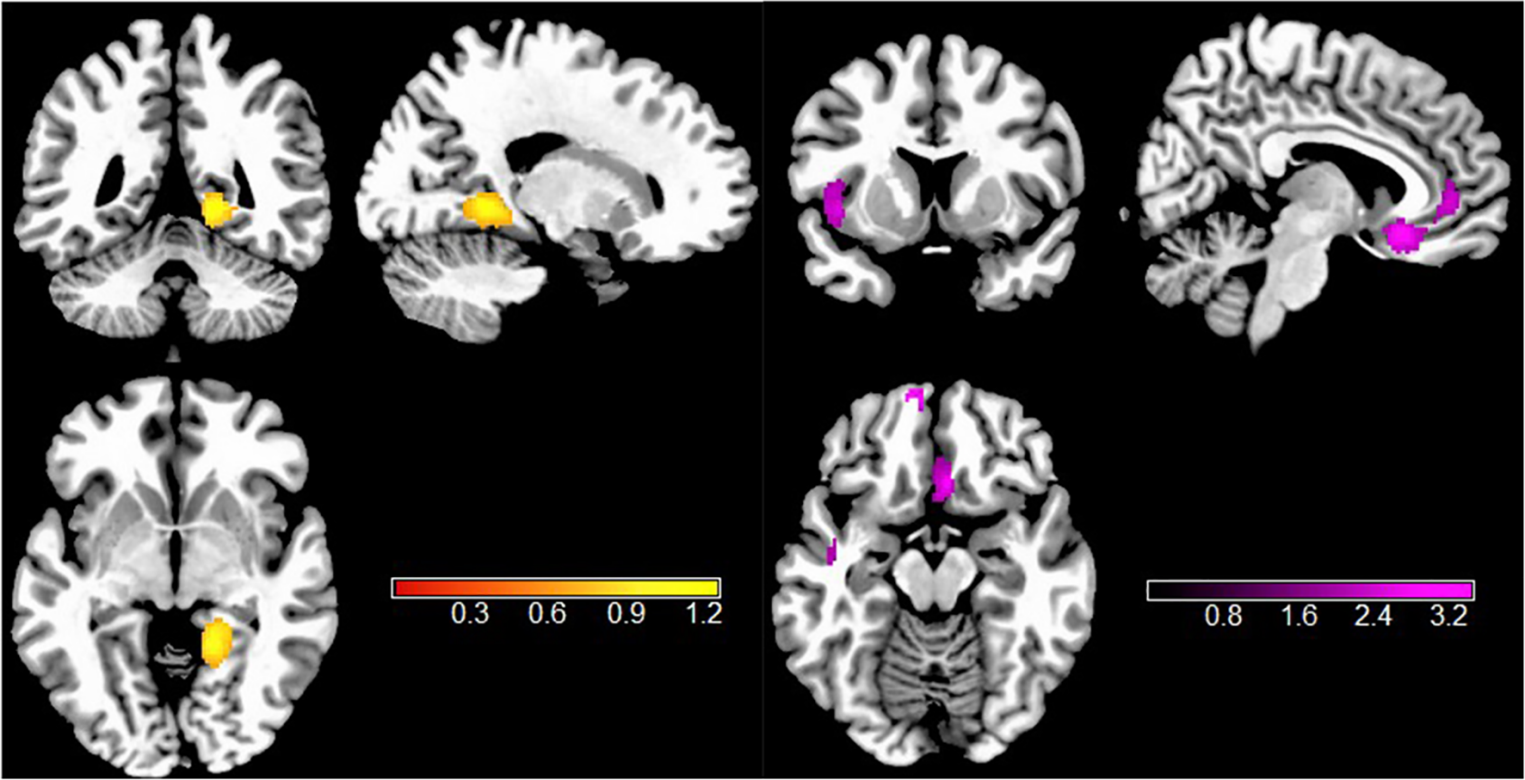
Results of VBM meta-analysis for individuals with TUD compared with HCs. Clusters are shown in the sagittal, axial, and coronal planes. Regions with GMV enlargement are shown in yellow, and GMV reductions are displayed in purple. VBM, voxel-based morphometry; TUD, tobacco use disorder; HCs, healthy controls; GMV, gray matter volume.

Gathering across all VBM studies, individuals with TUD showed significantly decreased GMV in the left superior temporal gyrus (STG.L), right superior frontal gyrus, medial orbital (SFG.R), right anterior cingulate/paracingulate gyrus (ACC.R), left superior frontal gyrus, medial orbital (SFG.L), and right anterior thalamic region (THA.R). In addition, increased GMV was found in the right lingual gyrus (LING.R) (**Figure 2**, **Table 2**).

**Table 2:** Brain locations of rs-fMRI and VBM differences in individuals with TUD compared to HCs.

Meta-analysis	Anatomical region	MNI coordinate	Number of voxels	SDM-z	p-Value	Jack-knife sensitivity	BA
1. rs-fMRI results							
TUD>HC	Right cerebellum, crus II	28, −74, −40	719	1.317	0.000799954	33 out of 35	NA
	Left superior frontal gyrus, dorsolateral	−14, 64, 16	121	1.356	0.000660598	34 out of 35	10
	Left inferior parietal gyrus	−42, −54, 56	30	1.125	0.002291381	33 out of 35	40
	Left supplementary motor area	−6, 16, 62	12	1.094	0.002802312	31 out of 35	12
TUD<HC	Right gyrus rectus	4, 30, −20	2143	−2.338	0.000015497	35 out of 35	11
	Right superior frontal gyrus, dorsolateral	16, 52, 32	215	−1.661	0.000686407	34 out of 35	9
	Right middle frontal gyrus	46, 44, 6	153	−1.624	0.000836074	34 out of 35	45
	Left inferior frontal gyrus, triangular part	−50, 26, 8	36	−1.401	0.002616525	33 out of 35	45
2. VBM results							
TUD<HC	Left superior temporal gyrus	−42, −20, −2	635	−3.415	0.000072241	28 out of 28	48
	Right superior frontal gyrus, medial orbital	6, 24, −14	185	−3.199	0.000211596	27 out of 28	11
	Right anterior cingulate/paracingulate gyrus	8, 46, 2	91	−2.881	0.000887632	27 out of 28	10
	Left superior frontal gyrus, medial orbital	−10, 62, −14	85	−3.052	0.000392199	26 out of 28	11
	Right anterior thalamic projections	38, 50, −4	38	−2.776	0.001331508	26 out of 28	NA
TUD>HC	Right lingual gyrus	18, −46, −2	324	1.245	0.000087738	26 out of 28	27
**3. Multimodal analysis**	Right lingual gyrus	14, −40, −4	341	1.000	—	—	27

TUD, tobacco use disorder; HC, healthy controls; MNI, Montreal Neurological Institute; SDM, Seed-based Mapping; BA, Brodmann area; rs-fMRI, resting-state functional magnetic resonance imaging; VBM, voxel-based morphometry; NA, not available.

#### rs-fMRI meta-analysis in TUD

**Figure 3:**
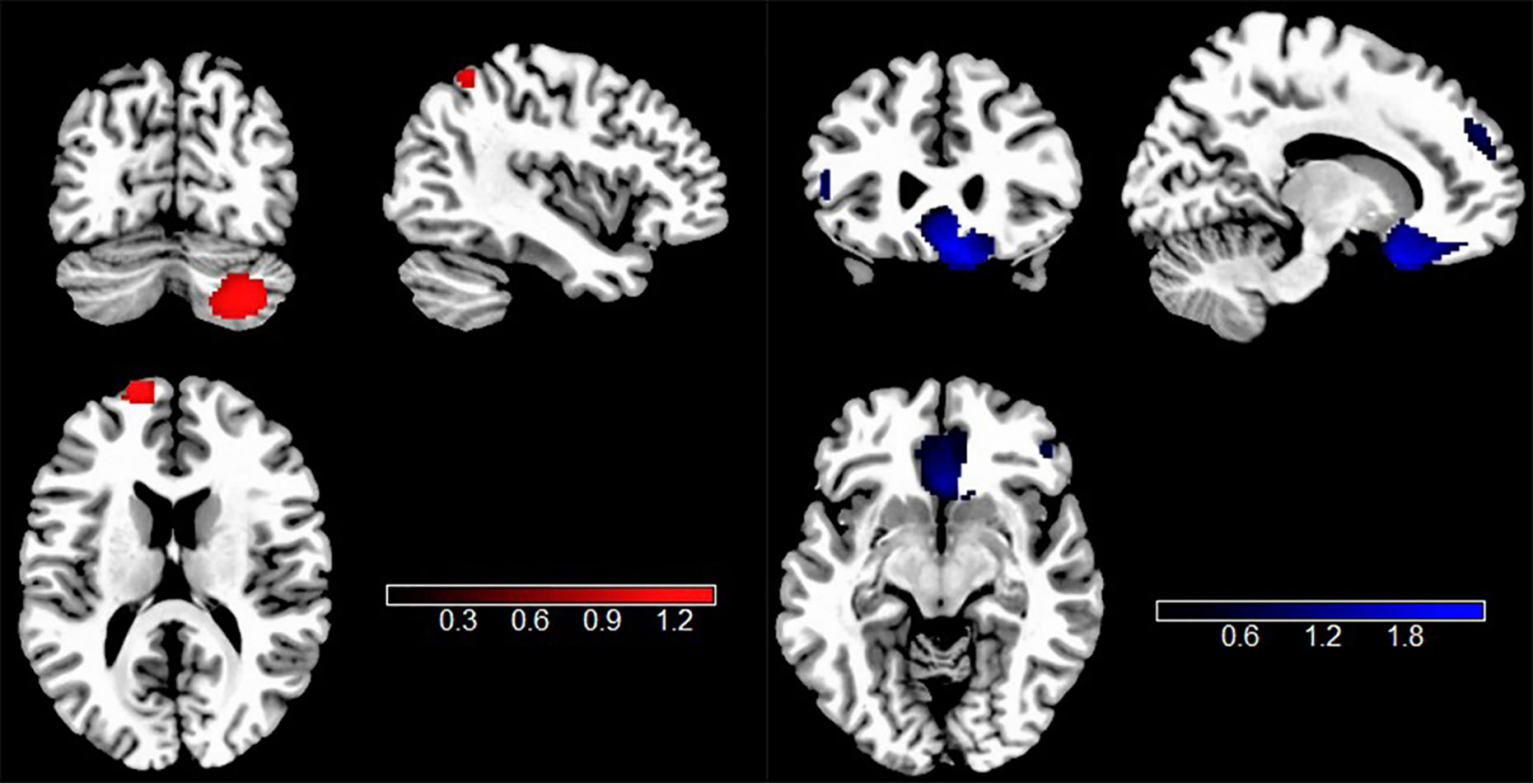
Results of rs-fMRI meta-analysis for individuals with TUD compared with HCs. Clusters are shown in the sagittal, axial, and coronal planes. Regions with rs-fMRI hyperactivity are shown in red, and those with rs-fMRI hypoactivity are displayed in blue. rs-fMRI, resting-state functional magnetic resonance imaging; TUD, tobacco use disorder; HCs, healthy controls.

According to all rs-fMRI meta-analyses, intrinsic neural activity was significantly different in individuals with TUD. Compared to HCs, individuals with TUD had significantly increased intrinsic function in the right cerebellum crus2, left superior frontal gyrus, dorsolateral (SFGdor.L), left inferior parietal gyrus (IPG.L), and left supplementary motor area (SMA.L) but decreased intrinsic function in the right gyrus rectus (REC.R), right superior frontal gyrus, dorsolateral (SFGdor.R), right middle frontal gyrus (MFG.R), and left inferior frontal gyrus in the triangular part (IFGtriang.L) (**Figure 3**, **Table 2**).

#### Multimodal VBM and rs-fMRI analyses in TUD

**Figure 4:**
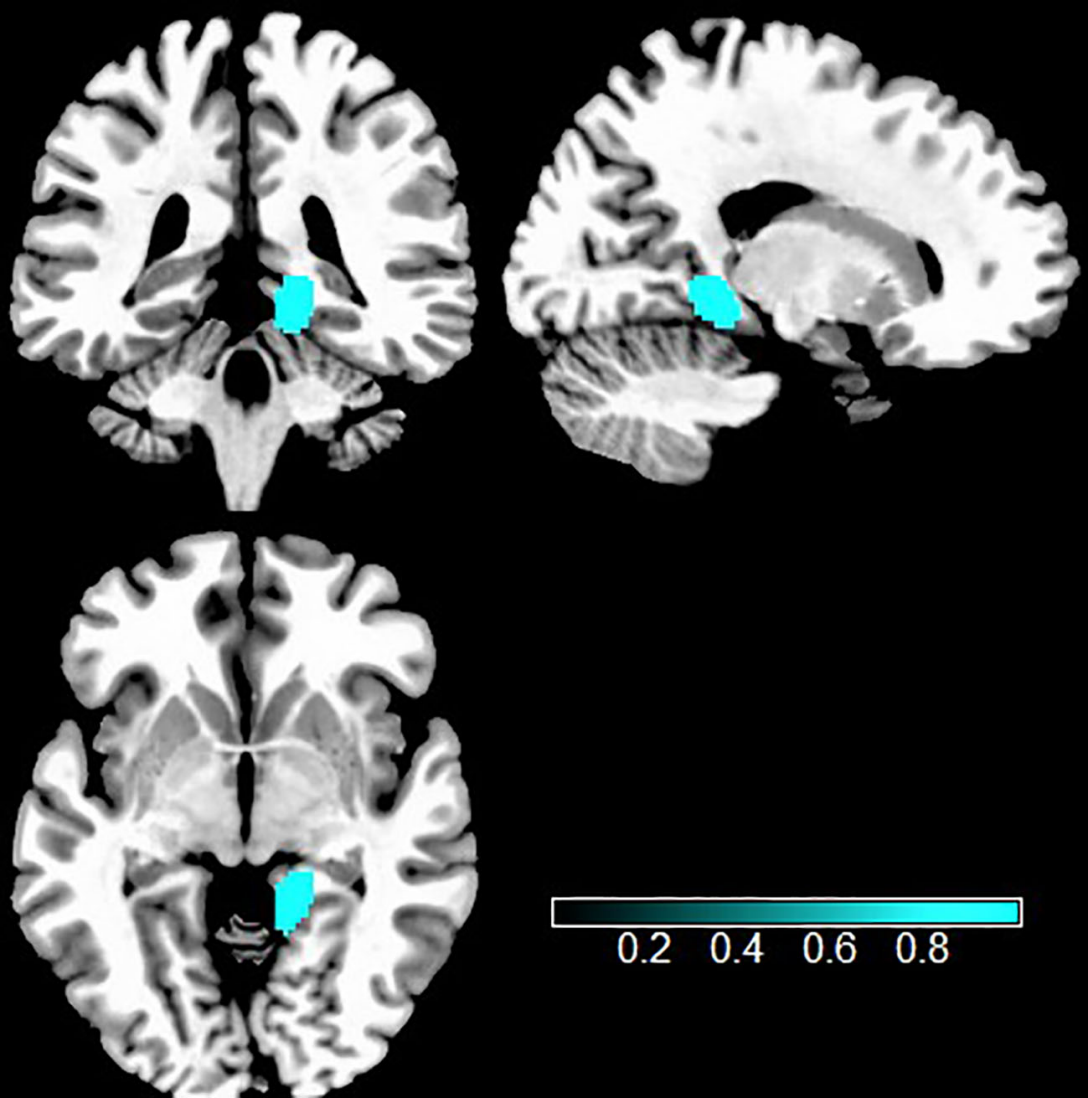
Multimodal analysis in TUD. Teal indicates increased GMV and intrinsic function. TUD, tobacco use disorder; GMV, gray matter volume.

The study of the convergence between two meta-analyses discovered that individuals with TUD showed both increased GMV and hyperactivity in the right lingual gyrus, compared with HCs (**Figure 4**, **Table 2**).

### Analysis of reliability

Jackknife sensitivity analysis indicated that the majority of the regions displayed high reliability. Out of the 37 rs-fMRI datasets, the REC.R was replicated in 35 combinations. The SFGdor.L, SFGdor.R, and MFG.R remained in 34 combinations. The right cerebellum crus2, IPG.L, and IFGtriang were replicated in 33 combinations. The SMA.L was replicated in 31 combinations.

Out of the 28 VBM datasets, the STG.L was replicated in 28 combinations. The SFG.R and ACC.R remained in 27 combinations. The LING.R, SFG.L, and THA.R were replicated in 26 combinations.

### Publication bias

Egger’s tests were performed to investigate potential publication bias. The results of Egger’s tests showed that there was no significant publication bias (p > 0.05, Bonferroni corrected) (32).

### Subgroup analysis

#### For methods

The subgroup analysis of FC datasets (n = 20) showed that individuals with TUD showed increased values of FC in the left inferior parietal gyrus and left supplementary motor area while decreased values of FC in the right caudate nucleus, left inferior frontal gyrus, and right cuneus cortex.

The subgroup analysis of ReHo datasets (n = 7), compared to HCs, showed that individuals with TUD showed increased values of ReHo in the right cerebellum crus2 and left superior parietal gyrus while decreased values of ReHo in the right middle frontal gyrus, right superior frontal gyrus dorsolateral, right superior frontal gyrus in the orbital part, and left cerebellum crus1.

The subgroup analysis of ALFF datasets (n = 4) revealed that individuals with TUD had increased values of ALFF in the left superior frontal gyrus, dorsolateral, and left middle frontal gyrus and decreased ALFF values in the left cerebellum.

**Figure 5:**
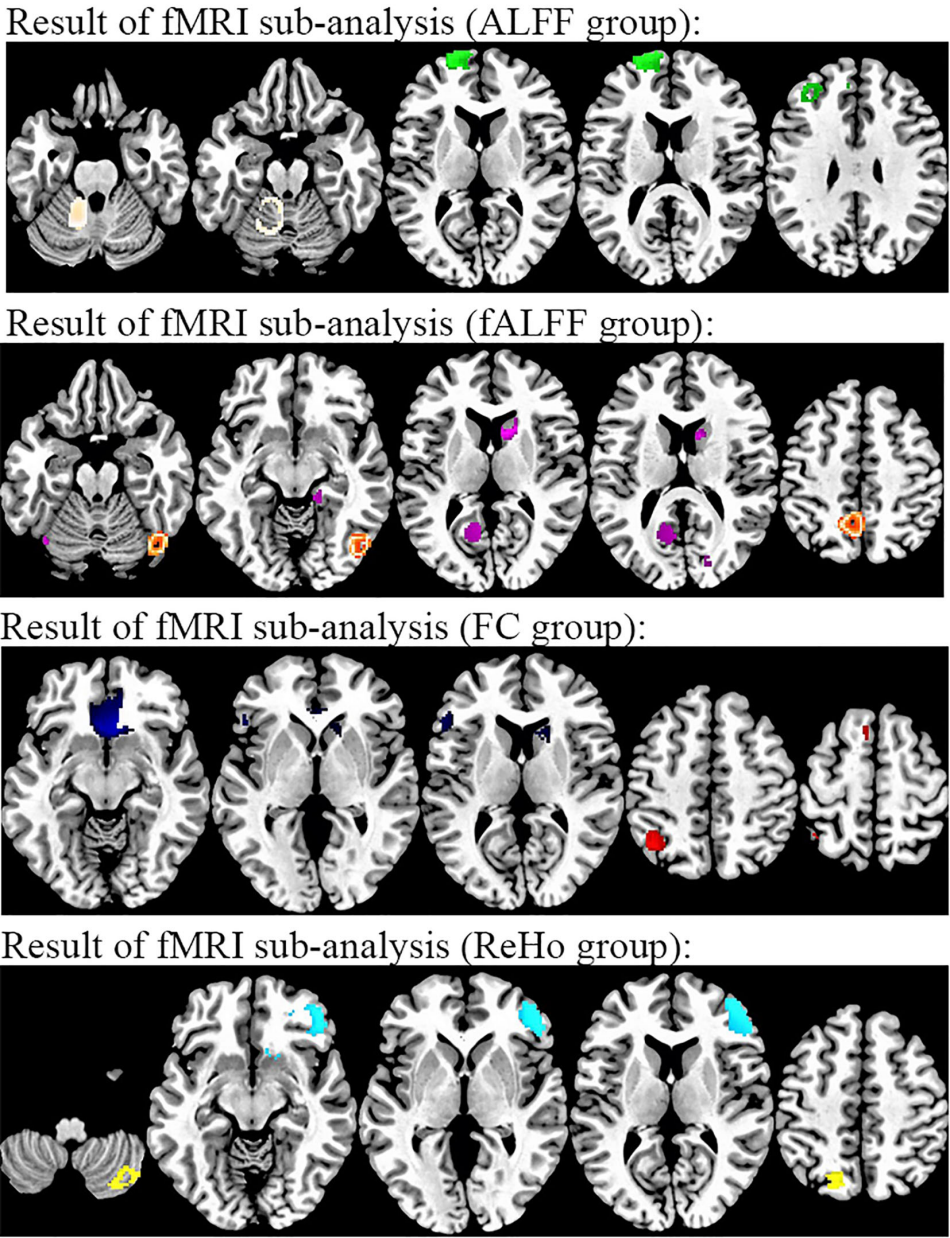
Results of subgroup analysis of rs-fMRI from methods. From top to bottom, individuals with TUD in the ALFF subgroup relative to healthy controls (white, increased in TUD; green, decreased in TUD), individuals with TUD in the fALFF subgroup relative to healthy controls (orange, increased in TUD; purple, decreased in TUD), individuals with TUD in the FC subgroup relative to healthy controls (red, increased in TUD; blue, decreased in TUD), and individuals with TUD in ReHo relative to healthy controls (yellow, increased in TUD; cyan, decreased in TUD). rs-fMRI, resting-state functional magnetic resonance imaging; ALFF, amplitude of low-frequency fluctuation; TUD, tobacco use disorder; fALFF, fractional amplitude of low-frequency fluctuation; FC, functional connectivity; ReHo, regional homogeneity.

The subgroup analysis of fALFF datasets (n = 4) revealed that individuals with TUD had increased fALFF values in the right caudate nucleus, left calcarine fissure, and left fusiform gyrus and decreased values of fALFF in the left precuneus and right inferior temporal gyrus (**Figure 5** and **Supplementary Table S2**).

#### For age

##### rs-fMRI groups in TUD

The subgroup analysis of adult datasets (n = 28) was in general consistent with the results of the pooled analysis. However, in the adult group, the intrinsic function of the left supplementary motor area, right rectus gyrus, and left inferior frontal gyrus was not detected. Compared to the pooled analysis, the decreased intrinsic function was found in the left precuneus. The results of the subgroup analysis of adolescent datasets (n = 9) indicated reduced intrinsic function in the left anterior cingulate gyrus, the left thalamus, and the right caudate nucleus compared to the pooled analysis.

##### VBM groups in TUD

**Figure 6:**
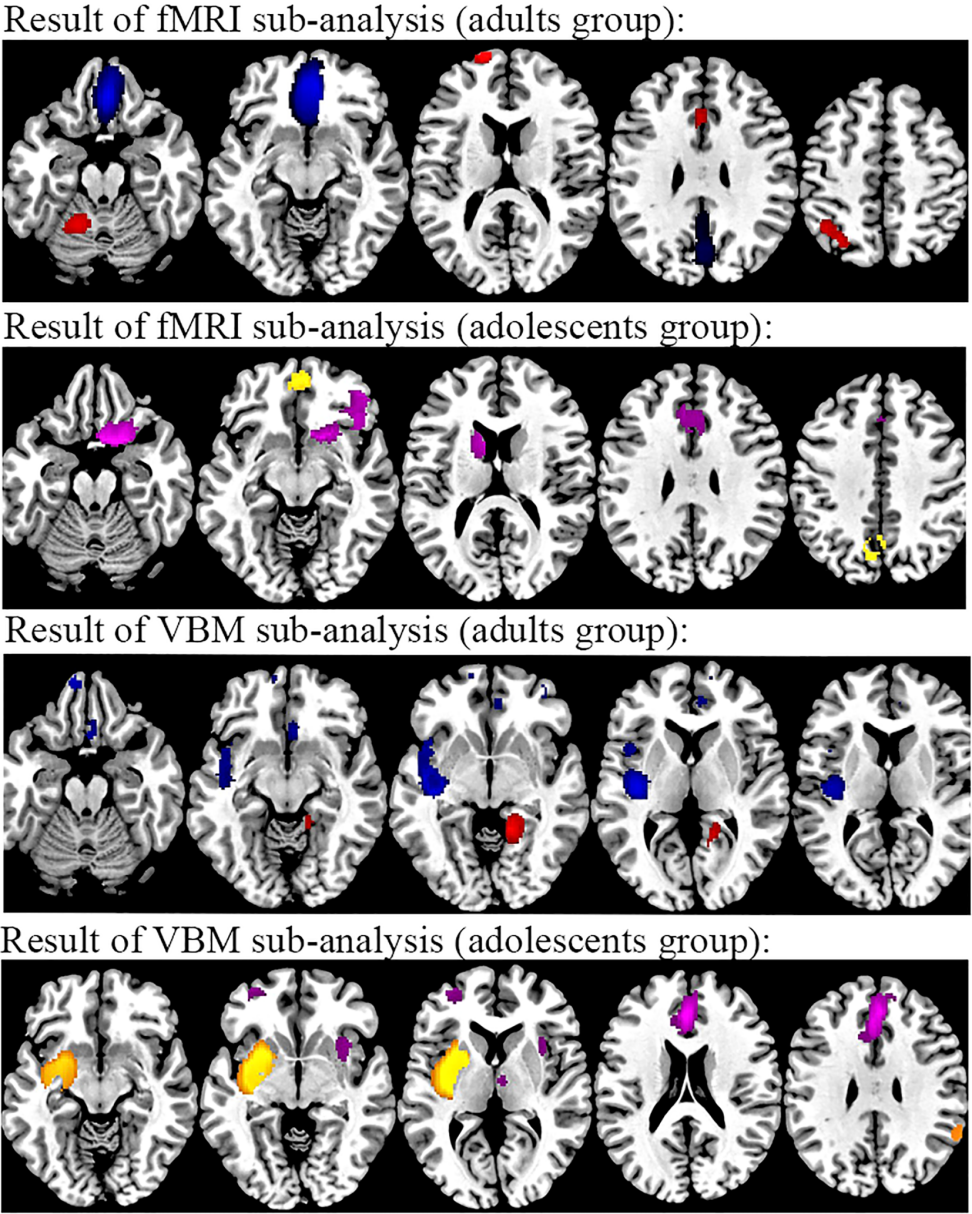
Results of subgroup analysis of rs-fMRI and VBM from age. From top to bottom, rs-fMRI results of individuals with TUD in the adult subgroup compared to healthy controls (red, increased in TUD; blue, decreased in TUD), rs-fMRI results of individuals with TUD in the adolescent subgroup compared to healthy controls (yellow, increased in TUD; purple, decreased in TUD), VBM results of individuals with TUD in the adult subgroup compared to healthy controls (red, increased in TUD; blue, decreased in TUD), and rs-fMRI results of individuals with TUD in the adolescent subgroup compared to healthy controls (yellow, increased in TUD; purple, decreased in TUD). rs-fMRI, resting-state functional magnetic resonance imaging; VBM, voxel-based morphometry; TUD, tobacco use disorder.

The subgroup analysis of adult datasets (n = 24) was consistent with the results of the pooled analysis, except for a decreased GMV in the left insula. The results of subgroups of adolescent datasets (n = 4) indicated increased GMV in the left striatum and right parahippocampal gyrus and decreased GMV in the right lenticular nucleus compared to the pooled analysis (**Figure 6** and **Supplementary Table S3**).

## Discussion

This study integrated 35 rs-fMRI studies and 25 VBM studies to identify structural and functional alterations in individuals with TUD, which employed the anisotropic seed-based d mapping (AES-SDM) method to investigate the neural mechanisms underlying TUD. The rs-fMRI results found that individuals with TUD had increased intrinsic function in the right cerebellum, SFGdor.L, IPG.L, and SMA.L and decreased intrinsic function in the SFGdor.R, MFG.R, IFGtriang.L, and REC.R. The VBM results showed that individuals with TUD showed decreased GMV in the STG.L, SFG.R, ACC.R, SFG.L, and THA.R and increased GMV in the LING.R. Following multimodal analysis, we observed an abnormal augmentation in GMV and function in the LING.R. This comprehensive approach allows for a more in-depth exploration of the structural and functional alterations observed in individuals with TUD and provides valuable insights into the underlying neural mechanisms contributing to this disorder.

Through the meta-analyses of VBM and rs-fMRI, as a significant part of the prefrontal cortex, the MFG.R, the IFG.L, and the SFG.B were found to have abnormal patterns of structural and functional alternations, which are considered to be associated with impaired motor function and cognition (38, 43). The pooled meta-analysis across various addiction-related disorders also observed similar patterns (72, 73). In addition, a study investigating regional brain perfusion levels in chronic smokers discovered that individuals with TUD showed significant deficits in multiple regions: anterior frontal cortices, bilateral superior temporal gyrus, and posterior cingulate (74). Among these brain regions, the dorsolateral superior frontal gyrus (DLSFC) has been widely recognized for its involvement in working memory, attention allocation, and cognitive manipulation (58, 60), and the MFG has been identified as a crucial brain region responsible for the inhibitory control of neural activity (75). Furthermore, the IFG is also considered to be involved in response inhibition and top-down regulatory control (76). Structure and function within this region may contribute to deficits in inhibitory control observed in individuals with TUD. Previous studies have provided evidence suggesting that tobacco has an impact on the resting-state networks (RSNs), which are closely associated with the occurrence and development of tobacco use disorder (44–46, 77). In terms of brain networks, these differentiated brain regions are simultaneously part of the executive control network (ECN). Neuroimaging studies have consistently shown the significant involvement of the ECN in top-down cognitive control processes (49). The ECN is associated with working memory and cognitive behavior (78). It is responsible for regulating attention and decision-making processes, as well as facilitating goal-directed behaviors (79). Dysregulation of the ECN has been observed in various neuropsychiatric disorders, including TUD, and may contribute to the cognitive impairments and difficulties in behavioral control experienced by individuals with TUD (80). Based on these findings, the aforementioned aberrant brain network activity may be involved in the pathological mechanisms of TUD and also confirms previous VBM and fMRI studies of TUD. Currently, transcranial magnetic stimulation (TMS) is a non-invasive method that is widely utilized for the treatment of numerous neuropsychiatric disorders (81). Researchers have discovered that repetitive transcranial magnetic stimulation targeting the dorsolateral prefrontal cortex has been shown to significantly reduce nicotine craving in response to cues (82). Therefore, in the future, we will continue to focus on the alternations in the structure and function of the prefrontal cortex (PFC) after the use of TMS in individuals with TUD compared to the pre-treatment period in order to determine the reliability and clinical value of the existing studies.

The increased intrinsic function of the IPG.L with increased GMV in the STG.L found in our study should also be noted. The IPG is responsible for receiving input from external sensory stimuli and may enable the transformation of internal goals to external actions through the intentional initiation of action interrelated with the mechanisms of primacy sensorimotor transformation (41), which is essential for the successful execution of planned actions in response to the surrounding environment (83). The STG is responsible for the processing and expression of visual information and has been found to play an important role in emotion regulation (35). Meanwhile, the IPG and STG are also vital components in the default mode network (DMN). The DMN is composed of several brain regions that are consistently active during rest and deactivated during external goal-directed tasks. The DMN has been implicated in a range of cognitive processes, including self-referential processing, autobiographical memory, social cognition, and mind-wandering (55, 84). Additionally, studies have found aberrant DMN connectivity in individuals with bipolar disorder, implicating its involvement in mood regulation and emotional processing (85, 86). In recent years, numerous studies have demonstrated the involvement of the DMN in addiction-related behaviors (77). This is consistent with our meta-analysis results. Structural and functional disorders of the IPG and the STG suggested functional imbalance within the DMN. This may result in an impaired capacity to resist nicotine, contributing to difficulties in overcoming tobacco addiction.

Traditionally, the anterior cingulate gyrus has been primarily involved in decision-making and the regulation of impulsive behaviors (64). A comparative study revealed that GABA concentrations in the ACC were associated with neurocognition, decision-making, and impulsivity in individuals with TUD (53, 87) and decreased intrinsic function of the ACC and reduced craving for cigarettes in individuals with TUD following bupropion hydrochloride exposure (88). In addition, the right ACC and the left SMA form the largest “regulator” of the human brain: the salience network (SN) (42, 62). It regulates the DMN and ECN to switch flexibly when processing internal and external information, thus ensuring the healthy and normal functioning of our brain (89). All in all, from this meta-analysis, we discovered that while the disputation of regions exhibiting structural and functional alternations is sporadic, the majority of them are components of the DMN, ECN, and SN. Our results further support the theory that the triple network plays a crucial role in TUD. In the future, we encourage researchers to focus not only on the development of new technologies but also on the less researched area of abnormal connectivity patterns of the classical tri-network and the effects on cognition, decision-making, and control in individuals with mental disorders.

In contrast to prior meta-analyses focusing on a single aspect in TUD, the current study pooling two meta-analyses for multimodal analysis aims to discover increased GMV and intrinsic function in the LING.R. Previous results have focused on the familiar reward pathways: the mesolimbic-frontal pathway and the mesolimbic-striatal pathway (56, 66, 67). However, this effect frequently occurs in the early stages of addiction, when large amounts of dopamine are released through the ventral tegmental area (VTA), acting on dopamine receptors in the prefrontal cortex, putamen, or caudate nucleus, inducing behaviors (52, 57, 90). As the research progressed, it became clear that individuals with TUD have attentional biases and visual information integration deficits (69). In fact, the LING.R is part of the occipital lobe (61). Anatomically, it is located between the calcarine and collateral sulcus. A study investigating the neural responses of adolescents after observing e-cigarette advertisements found a significant intrinsic function of visual attention areas (left lingual gyrus/fusiform gyrus) (91) and an increased desire for smoking in adolescents who had difficulty resisting the temptation to smoke cigarettes. Therefore, the role of the lingual gyrus in TUD deserves further in-depth exploration.

The role of the thalamus in drug self-administration behavior cannot be ignored as an important component of the impaired response inhibition and significant attribution (IRSA) model (92). The thalamus is involved in sensory processing, memory, and motivated behaviors (47, 65, 68). However, despite our agreement, the diversity of thalamus functions depends on interactions between and within the intrinsic nuclei of the thalamus. A recent study used state-of-the-art expression profiling to propose the unique concept of thalamus brain networks (93). Therefore, more emphasis should be placed on the internal nuclei of the thalamus. We will further explore the complex relationship between the nuclei of the thalamus and the intrinsic pathogenesis of TUD using subcortical volume segmentation or fiber tracking. Traditionally associated with coordinating movement and maintaining balance, recent studies have revealed that the cerebellum also has extensive connectivity with various regions of the brain (39, 63, 94). Such connectivity suggests that the cerebellum is involved in higher-order cognitive processes, including motor planning, language processing, spatial perception, and executive functions (54, 70, 95). A positron emission tomography (PET) study revealed increased regional cerebral blood flow (CBF) in the cerebellum of individuals with TUD when compared to HCs (96). At the same time, the thalamus and cerebellum are regions with high nicotinic acetylcholine receptor (nAChR) density in the brain (59, 97). Then, under the actions of the harmful substance nicotine, the structure and function are bound to be damaged to a certain extent. However, this causal relationship between the behavior of individuals with TUD and deficits in differential brain regions needs to be further explored.

In addition, we observed increased intrinsic function in the REC.R, which has received less attention due to its being less recognized as a significant component in the addiction circuit. However, the decrease of intrinsic function in PFC has been mentioned in numerous studies (36, 40, 54, 71), while the REC.R only discovers one direct corresponding study in the original study (34). We speculated that this may be due to the utilization of the SDM software for meta-analysis, which allows for more precise and detailed coordinate positioning. However, some studies have reported more general coordinates, such as a larger range of the medial superior frontal gyrus or anterior cingulate, rather than providing more precise coordinates, which may result in some loss of spatial specificity (15, 48, 51).

Finally, subgroup analyses were conducted to explore the potential sources of heterogeneity in this study. Methodologically, the subgroup analysis revealed that the MFG.R and left dorsolateral superior frontal gyrus (DLSFG.L) showed a dependence on ALFF, the right cerebellum crus2 and right dorsolateral superior frontal gyrus (DLSFG.R) exhibited a dependence on ReHo, while the IPG.L, SMA.L, and IFG.L demonstrated a dependence on FC. However, it is worth noting that no significant heterogeneity originating from ALFF was observed in this study, which may be attributed to the relatively small sample size. Furthermore, in terms of age, subgroups of adults are generally consistent with pooled analysis results in rs-fMRI and VBM. This not only indicated the reliability of our meta-analysis results but also demonstrated the importance of these brain regions in TUD. However, the subgroup of adults had decreased intrinsic function in the left precuneus and decreased GMV in the left insula compared with the pooled analysis. The subgroup of adolescents had decreased intrinsic function in the right caudate nucleus and increased GMV in the left striatum, right parahippocampal gyrus, and right lenticular nucleus. This subgroup analysis also confirms the previous conjecture that the initial stage of the addiction process relies on the frontal-thalamic-striatal circuitry to mediate the initial reinforcing effects (called reward deficiency syndrome) and causes deficits in the parahippocampal gyrus, which is responsible for memory functions (21, 50). With increasing years of smoking, structural or functional decline emerges in brain regions associated with vision, and individuals with TUD slowly develop attentional deficits and selective bias, which contributes to the habituation of tobacco smoking behavior over time (26). Undoubtedly, continual research is essential to substantiate our hypothesis further.

## Limitation

Despite this meta-analysis providing some significant discoveries, several limitations still prevented a thorough examination of the findings. First, previous research has demonstrated gender differences in individuals with TUD (37). However, the entire sample directly included in our study (male majority) did not identify potential neurological differences in individuals with TUD from a gender perspective. Second, the differences in MRI scans, slice thickness, and statistical thresholding may have led to inconsistent results. Third, we only use peak coordinates and effect sizes from studies, which may have ignored some alterations in raw statistical brain maps. Fourth, the adolescent subgroup is too small to provide reliable conclusions. In the future, we will expand our sample size and continue to focus on functional and structural brain changes in adolescents with TUD and timely updates to the current meta-analysis. Finally, further longitudinal studies are needed to demonstrate whether alterations in this brain can serve as targets for treating nicotine dependence.

## Conclusion

According to pooled analysis, individuals with TUD exhibit altered patterns of structural and functional abnormalities. Through the integration of VBM and rs-fMRI studies, the significance of the DMN, ECN, and SN in the progression of tobacco addiction was demonstrated. In addition, multimodal analysis revealed structural and functional abnormalities in the right lingual gyrus. These alterations may be closely associated with the pathophysiological mechanism of tobacco addictions, which may contribute to the diagnosis and treatment of individuals with TUD.

## Data Availability

The original contributions presented in the study are included in the article/**Supplementary Material**. Further inquiries can be directed to the corresponding author.

## Supplementary material

Supplementary Figure 1 Funnel plots for significant results.

Supplementary Table 1 Clinical characteristics and Technical details of rs-fMRI and VBM studies in TUD.

Supplementary Table 2 Brain locations of FC, ReHo, ALFF, and fALFF differences in individuals with TUD compared to HCs.

Supplementary Table 3 Subgroup analysis results for VBM and rs-fMRI studies in TUD (adults group and adolescents group).

## References

[1] Schroeder SA . New evidence that cigarette smoking remains the most important health hazard. N Engl J Med. (2013) 368:389–90. doi: 10.1056/NEJMe1213751 23343069

[2] Kalivas PW Volkow ND . The neural basis of addiction: a pathology of motivation and choice. Am J Psychiatry. (2005) 162:1403–13. doi: 10.1176/appi.ajp.162.8.1403 16055761

[3] Durojaye E . The WHO tobacco convention: A new dawn in the implementation of international health instrument? Comment on “The legal strength of international health instruments - what it brings to global health governance? Int J Health Policy Manag. (2018) 7:189–91. doi: 10.15171/ijhpm.2017.70 PMC5819380 29524944

[4] Tyas SL White LR Petrovitch H Webster Ross G Foley DJ Heimovitz HK . Mid-life smoking and late-life dementia: the Honolulu-Asia Aging Study. Neurobiol Aging. (2003) 24:589–96. doi: 10.1016/S0197-4580(02)00156-2 12714116

[5] Campos MW Serebrisky D Castaldelli-Maia JM . Smoking and cognition. Curr Drug Abuse Rev. (2016) 9:76–9. doi: 10.2174/1874473709666160803101633 27492358

[6] Jakab Z . Smoking and pregnancy. Acta Obstet Gynecol Scand. (2010) 89:416–17. doi: 10.3109/00016341003732349 20367425

[7] Zhuang YL Gamst AC Cummins SE Wolfson T Zhu SH . Comparison of smoking cessation between education groups: findings from 2 US National Surveys over 2 decades. Am J Public Health. (2015) 105:373–9. doi: 10.2105/AJPH.2014.302222 PMC4289455 25521868

[8] Gray NJ . Nicotine yesterday, today, and tomorrow: a global review. Nicotine Tob Res. (2014) 16:128–36. doi: 10.1093/ntr/ntt171 24191982

[9] Zhu SH Lee M Zhuang YL Gamst A Wolfson T . Interventions to increase smoking cessation at the population level: how much progress has been made in the last two decades. Tob Control. (2012) 21:110–8. doi: 10.1136/tobaccocontrol-2011-050371 PMC3446870 22345233

[10] Brody AL Mandelkern MA Jarvik ME Lee GS Smith EC Huang JC . Differences between smokers and nonsmokers in regional gray matter volumes and densities. Biol Psychiatry. (2004) 55:77–84. doi: 10.1016/S0006-3223(03)00610-3 14706428

[11] Liao Y Tang J Liu T Chen X Hao W . Differences between smokers and non-smokers in regional gray matter volumes: a voxel-based morphometry study. Addict Biol. (2012) 17:977–80. doi: 10.1111/j.1369-1600.2010.00250.x 20731627

[12] Zorlu N Cropley VL Zorlu PK Delibas DH Adibelli ZH Baskin EP . Effects of cigarette smoking on cortical thickness in major depressive disorder. J Psychiatr Res. (2017) 84:1–8. doi: 10.1016/j.jpsychires.2016.09.009 27669406

[13] Peng P Li M Liu H Tian YR Chu SL Van-Halm-Lutterodt N . Brain structure alterations in respect to tobacco consumption and nicotine dependence: A comparative voxel-based morphometry study. Front Neuroanat. (2018) 12:43. doi: 10.3389/fnana.2018.00043 29881337 PMC5978277

[14] Fritz HC Wittfeld K Schmidt CO Domin M Grabe HJ Hegenscheid K . Current smoking and reduced gray matter volume-a voxel-based morphometry study. Neuropsychopharmacology. (2014) 39:2594–600. doi: 10.1038/npp.2014.112 PMC4207339 24832823

[15] Qiu X Han X Wang Y Ding W Sun Y Zhou Y . Interaction between smoking and internet gaming disorder on spontaneous brain activity. Front Psychiatry. (2020) 11:586114. doi: 10.3389/fpsyt.2020.586114 33343420 PMC7744462

[16] Zhang T Luo X Zeng Q Fu Y Li Z Li K . Effects of smoking on regional homogeneity in mild cognitive impairment: A resting-state functional MRI study. Front Aging Neurosci. (2020) 12:572732. doi: 10.3389/fnagi.2020.572732 33328955 PMC7717978

[17] Yu R Zhao L Tian J Qin W Wang W Yuan K . Regional homogeneity changes in heavy male smokers: a resting-state functional magnetic resonance imaging study. Addict Biol. (2013) 18:729–31. doi: 10.1111/j.1369-1600.2011.00359.x 21812873

[18] Wang C Shen Z Huang P Yu H Qian W Guan X . Altered spontaneous brain activity in chronic smokers revealed by fractional ramplitude of low-frequency fluctuation analysis: a preliminary study. Sci Rep. (2017) 7:328. doi: 10.1038/s41598-017-00463-7 28336919 PMC5428464

[19] Qian W Huang P Shen Z Wang C Yang Y Zhang M . Brain gray matter volume and functional connectivity are associated with smoking cessation outcomes. Front Hum Neurosci. (2019) 13:361. doi: 10.3389/fnhum.2019.00361 31680913 PMC6803765

[20] Faulkner P Lucini Paioni S Kozhuharova P Orlov N Lythgoe DJ Daniju Y . Daily and intermittent smoking are associated with low prefrontal volume and low concentrations of prefrontal glutamate, creatine, myo-inositol, and N-acetylaspartate. Addict Biol. (2021) 26:e12986. doi: 10.1111/adb.12986 33274546

[21] Hanlon CA Owens MM Joseph JE Zhu X George MS Brady KT . Lower subcortical gray matter volume in both younger smokers and established smokers relative to non-smokers. Addict Biol. (2016) 21:185–95. doi: 10.1111/adb.2016.21.issue-1 PMC4326619 25125263

[22] Bi Y Yuan K Guan Y Cheng J Zhang Y Li Y . Altered resting state functional connectivity of anterior insula in young smokers. Brain Imaging Behav. (2017) 11:155–65. doi: 10.1007/s11682-016-9511-z 26843002

[23] Qiu T Xie F Zeng Q Shen Z Du G Xu X . Interactions between cigarette smoking and cognitive status on functional connectivity of the cortico-striatal circuits in individuals without dementia: A resting-state functional MRI study. CNS Neurosci Ther. (2022) 28:1195–204. doi: 10.1111/cns.13852 PMC9253779 35506354

[24] Shen Z Huang P Wang C Qian W Luo X Guan X . Altered function but not structure of the amygdala in nicotine-dependent individuals. Neuropsychologia. (2017) 107:102–07. doi: 10.1016/j.neuropsychologia.2017.11.003 29104080

[25] Akkermans SEA Luijten M van Rooij D Franken IHA Buitelaar JK . Putamen functional connectivity during inhibitory control in smokers and non-smokers. Addict Biol. (2018) 23:359–68. doi: 10.1111/adb.2018.23.issue-1 27917562

[26] Tan Q Li S Niu J Liu S Li Y Lu Y . Resting-state functional magnetic resonance imaging reveals overactivation of the habitual control brain system in tobacco dependence. Neuropsychiatr Dis Treat. (2021) 17:3753–68. doi: 10.2147/NDT.S334403 PMC8703225 34984003

[27] Wang C Bai J Wang C von Deneen KM Yuan K Cheng J . Altered thalamo-cortical resting state functional connectivity in smokers. Neurosci Lett. (2017) 653:120–25. doi: 10.1016/j.neulet.2017.05.038 28536051

[28] Wang C Shen Z Huang P Qian W Yu X Sun J . Altered spontaneous activity of posterior cingulate cortex and superior temporal gyrus are associated with a smoking cessation treatment outcome using varenicline revealed by regional homogeneity. Brain Imaging Behav. (2017) 11:611–18. doi: 10.1007/s11682-016-9538-1 26960945

[29] Cortese S Aoki YY Itahashi T Castellanos FX Eickhoff SB . Systematic review and meta-analysis: resting-state functional magnetic resonance imaging studies of attention-deficit/hyperactivity disorder. J Am Acad Child Adolesc Psychiatry. (2021) 60:61–75. doi: 10.1016/j.jaac.2020.08.014 32946973

[30] Radua J Mataix-Cols D Phillips ML El-Hage W Kronhaus DM Cardoner N . A new meta-analytic method for neuroimaging studies that combines reported peak coordinates and statistical parametric maps. Eur Psychiatry. (2012) 27:605–11. doi: 10.1016/j.eurpsy.2011.04.001 21658917

[31] Radua J Rubia K Canales-Rodríguez EJ Pomarol-Clotet E Fusar-Poli P Mataix-Cols D . Anisotropic kernels for coordinate-based meta-analyses of neuroimaging studies. Front Psychiatry. (2014) 5:13. doi: 10.3389/fpsyt.2014.00013 24575054 PMC3919071

[32] Sterne JA Sutton AJ Ioannidis JP Terrin N Jones DR Lau J . Recommendations for examining and interpreting funnel plot asymmetry in meta-analyses of randomised controlled trials. BMJ. (2011) 343:d4002. doi: 10.1136/bmj.d4002 21784880

[33] Sawyer SM Azzopardi PS Wickremarathne D Patton GC . The age of adolescence. Lancet Child Adolesc Health. (2018) 2:223–28. doi: 10.1016/S2352-4642(18)30022-1 30169257

[34] Chen X Wang Y Zhou Y Sun Y Ding W Zhuang Z . Different resting-state functional connectivity alterations in smokers and nonsmokers with Internet gaming addiction. BioMed Res Int. (2014) 2014:825787. doi: 10.1155/2014/825787 25506057 PMC4255056

[35] Zhou S Xiao D Peng P Wang SK Liu Z Qin HY . Effect of smoking on resting-state functional connectivity in smokers: An fMRI study. Respirology. (2017) 22:1118–24. doi: 10.1111/resp.2017.22.issue-6 28374936

[36] Ge X Sun Y Han X Wang Y Ding W Cao M . Difference in the functional connectivity of the dorsolateral prefrontal cortex between smokers with nicotine dependence and individuals with internet gaming disorder. BMC Neurosci. (2017) 18:54. doi: 10.1186/s12868-017-0375-y 28750618 PMC5530585

[37] Lin F Han X Wang Y Ding W Sun Y Zhou Y . Sex-specific effects of cigarette smoking on caudate and amygdala volume and resting-state functional connectivity. Brain Imaging Behav. (2021) 15:1–13. doi: 10.1007/s11682-019-00227-z 31898088

[38] Niu X Gao X Lv Q Zhang M Dang J Sun J . Increased spontaneous activity of the superior frontal gyrus with reduced functional connectivity to visual attention areas and cerebellum in male smokers. Front Hum Neurosci. (2023) 17:1153976. doi: 10.3389/fnhum.2023.1153976 37007679 PMC10063805

[39] Shen Z Huang P Wang C Qian W Yang Y Zhang M . Cerebellar gray matter reductions associate with decreased functional connectivity in nicotine-dependent individuals. Nicotine Tob Res. (2018) 20:440–47. doi: 10.1093/ntr/ntx168 PMC6251678 29065207

[40] Stoeckel LE Chai XJ Zhang J Whitfield-Gabrieli S Evins AE . Lower gray matter density and functional connectivity in the anterior insula in smokers compared with never smokers. Addict Biol. (2016) 21:972–81. doi: 10.1111/adb.2016.21.issue-4 PMC4654721 25990865

[41] Zhang T Zeng Q Li K Liu X Fu Y Qiu T . Distinct resting-state functional connectivity patterns of Anterior Insula affected by smoking in mild cognitive impairment. Brain Imaging Behav. (2023) 17:386–94. doi: 10.1007/s11682-023-00766-6 PMC10435406 37243752

[42] Yip SW Lichenstein SD Garrison K Averill CL Viswanath H Salas R . Effects of smoking status and state on intrinsic connectivity. Biol Psychiatry Cognit Neurosci Neuroimaging. (2022) 7:895–904. doi: 10.1016/j.bpsc.2021.02.004 33618016 PMC8373998

[43] Qiu T Zeng Q Luo X Xu T Shen Z Xu X . Effects of cigarette smoking on resting-state functional connectivity of the nucleus basalis of meynert in mild cognitive impairment. Front Aging Neurosci. (2021) 13:755630. doi: 10.3389/fnagi.2021.755630 34867281 PMC8638702

[44] Wang X Xue T Dong F Li Y Xie D Liu C . The changes of brain functional networks in young adult smokers based on independent component analysis. Brain Imaging Behav. (2021) 15:788–97. doi: 10.1007/s11682-020-00289-4 32314196

[45] Claus ED Weywadt CR . Resting-state connectivity in former, current, and never smokers. Nicotine Tob Res. (2020) 22:180–87. doi: 10.1093/ntr/nty266 30590742

[46] Zhang Y Li YL Cheng JL Dong AK Xu K Li YL . Resting-state network evaluation of chronic smokers by functional magnetic resonance imaging. Zhonghua Yi Xue Za Zhi. (2017) 97:3724–28. doi: 10.3760/cma.j.issn.0376-2491.2017.47.009 29325327

[47] Huang W King JA Ursprung WW Zheng S Zhang N Kennedy DN . The development and expression of physical nicotine dependence corresponds to structural and functional alterations in the anterior cingulate-precuneus pathway. Brain Behav. (2014) 4:408–17. doi: 10.1002/brb3.2014.4.issue-3 PMC4055191 24944870

[48] Chen H Mo S . Regional homogeneity changes in nicotine addicts by resting-state fMRI. PloS One. (2017) 12:e0170143. doi: 10.1371/journal.pone.0170143 28081226 PMC5231336

[49] Wu G Yang S Zhu L Lin F . Altered spontaneous brain activity in heavy smokers revealed by regional homogeneity. Psychopharmacol (Berl). (2015) 232:2481–9. doi: 10.1007/s00213-015-3881-6 25716308

[50] Tang J Liao Y Deng Q Liu T Chen X Wang X . Altered spontaneous activity in young chronic cigarette smokers revealed by regional homogeneity. Behav Brain Funct. (2012) 8:44. doi: 10.1186/1744-9081-8-44 22913365 PMC3511796

[51] Wen Z Han X Wang Y Ding W Sun Y Kang Y . Sex-dependent alterations of regional homogeneity in cigarette smokers. Front Psychiatry. (2022) 13:874893. doi: 10.3389/fpsyt.2022.874893 35546937 PMC9082268

[52] Liu H Luo Q Du W Li X Zhang Z Yu R . Cigarette smoking and schizophrenia independently and reversibly altered intrinsic brain activity. Brain Imaging Behav. (2018) 12:1457–65. doi: 10.1007/s11682-017-9806-8 29297153

[53] Gao X Zhang M Yang Z Niu X Zhou B Chen J . Nicotine addiction and overweight affect intrinsic neural activity and neurotransmitter activity: A fMRI study of interaction effects. Psychiatry Clin Neurosci. (2023) 77:178–85. doi: 10.1111/pcn.13516 36468828

[54] Chu S Xiao D Wang S Peng P Xie T He Y . Spontaneous brain activity in chronic smokers revealed by fractional amplitude of low frequency fluctuation analysis: a resting state functional magnetic resonance imaging study. Chin Med J (Engl). (2014) 127:1504–9. doi: 10.3760/cma.j.issn.0366-6999.20131608 24762597

[55] Gao X Zhang M Yang Z Niu X Chen J Zhou B . Explore the effects of overweight and smoking on spontaneous brain activity: Independent and reverse. Front Neurosci. (2022) 16:944768. doi: 10.3389/fnins.2022.944768 36312021 PMC9597461

[56] Conti AA . Chronic tobacco smoking, impaired reward-based decision-making, and role of insular cortex: A comparison between early-onset smokers and late-onset smokers. Front Psychiatry. (2022) 13:939707. doi: 10.3389/fpsyt.2022.939707 36090372 PMC9459116

[57] Conti AA . Neuroanatomical correlates of impulsive choices and risky decision making in young chronic tobacco smokers: A voxel-based morphometry study. Front Psychiatry. (2021) 12:708925. doi: 10.3389/fpsyt.2021.708925 34526922 PMC8435625

[58] Zhang M Gao X Yang Z Niu X Wang W Han S . Integrative brain structural and molecular analyses of interaction between tobacco use disorder and overweight among male adults. J Neurosci Res. (2023) 101:232–44. doi: 10.1002/jnr.v101.2 36333937

[59] Daniju Y Faulkner P Brandt K Allen P . Prefrontal cortex and putamen grey matter alterations in cannabis and tobacco users. J Psychopharmacol. (2022) 36:1315–23. doi: 10.1177/02698811221117523 PMC9716493 36112825

[60] Kunas SL Hilbert K Yang Y Richter J Hamm A Wittmann A . The modulating impact of cigarette smoking on brain structure in panic disorder: a voxel-based morphometry study. Soc Cognit Affect Neurosci. (2020) 15:849–59. doi: 10.1093/scan/nsaa103 PMC7543937 32734299

[61] Ye Y Zhang J Huang B Cai X Wang P Zeng P . Characterizing the structural pattern of heavy smokers using multivoxel pattern analysis. Front Psychiatry. (2020) 11:607003. doi: 10.3389/fpsyt.2020.607003 33613332 PMC7890259

[62] Chen Y Chaudhary S Wang W Li CR . Gray matter volumes of the insula and anterior cingulate cortex and their dysfunctional roles in cigarette smoking. Addict Neurosci. (2022) 1. doi: 10.1016/j.addicn.2021.100003 PMC10201991 37220533

[63] Cai Z Wang P Liu B Zou Y Wu S Tian J . To explore the mechanism of tobacco addiction using structural and functional MRI: a preliminary study of the role of the cerebellum-striatum circuit. Brain Imaging Behav. (2022) 16:834–42. doi: 10.1007/s11682-021-00546-0 34606038

[64] Bu L Yu D Su S Ma Y von Deneen KM Luo L . Functional connectivity abnormalities of brain regions with structural deficits in young adult male smokers. Front Hum Neurosci. (2016) 10:494. doi: 10.3389/fnhum.2016.00494 27757078 PMC5047919

[65] Franklin TR Wetherill RR Jagannathan K Johnson B Mumma J Hager N . The effects of chronic cigarette smoking on gray matter volume: influence of sex. PloS One. (2014) 9:e104102. doi: 10.1371/journal.pone.0104102 25090480 PMC4121321

[66] Gallinat J Meisenzahl E Jacobsen LK Kalus P Bierbrauer J Kienast T . Smoking and structural brain deficits: a volumetric MR investigation. Eur J Neurosci. (2006) 24:1744–50. doi: 10.1111/j.1460-9568.2006.05050.x 17004938

[67] Morales AM Lee B Hellemann G O’Neill J London ED . Gray-matter volume in methamphetamine dependence: cigarette smoking and changes with abstinence from methamphetamine. Drug Alcohol Depend. (2012) 125:230–8. doi: 10.1016/j.drugalcdep.2012.02.017 PMC3427723 22445480

[68] Wang K Yang J Zhang S Wei D Hao X Tu S . The neural mechanisms underlying the acute effect of cigarette smoking on chronic smokers. PloS One. (2014) 9:e102828. doi: 10.1371/journal.pone.0102828 25051341 PMC4106848

[69] Peng P Wang Z Jiang T Chu S Wang S Xiao D . Brain-volume changes in young and middle-aged smokers: a DARTEL-based voxel-based morphometry study. Clin Respir J. (2017) 11:621–31. doi: 10.1111/crj.12393 26404024

[70] Yu R . Regional grey and white matter changes in heavy male smokers. PloS One. (2011) 6:e27440. doi: 10.1371/journal.pone.0027440 22076160 PMC3208641

[71] Zhang X Salmeron BJ Ross TJ Geng X Yang Y Stein EA . Factors underlying prefrontal and insula structural alterations in smokers. Neuroimage. (2011) 54:42–8. doi: 10.1016/j.neuroimage.2010.08.008 PMC2962727 20699124

[72] Yao YW Liu L Ma SS Shi XH Zhou N Zhang JT . Functional and structural neural alterations in Internet gaming disorder: A systematic review and meta-analysis. Neurosci Biobehav Rev. (2017) 83:313–24. doi: 10.1016/j.neubiorev.2017.10.029 29102686

[73] Spindler C Trautmann S Alexander N Bröning S Bartscher S Stuppe M . Meta-analysis of grey matter changes and their behavioral characterization in patients with alcohol use disorder. Sci Rep. (2021) 11:5238. doi: 10.1038/s41598-021-84804-7 33664372 PMC7933165

[74] Durazzo TC Meyerhoff DJ Murray DE . Comparison of regional brain perfusion levels in chronically smoking and non-smoking adults. Int J Environ Res Public Health. (2015) 12:8198–213. doi: 10.3390/ijerph120708198 PMC4515717 26193290

[75] Marek S Dosenbach NUF . The frontoparietal network: function, electrophysiology, and importance of individual precision mapping. Dialogues Clin Neurosci. (2018) 20:133–40. doi: 10.31887/DCNS.2018.20.2/smarek PMC6136121 30250390

[76] Zilverstand A Huang AS Alia-Klein N Goldstein RZ . Neuroimaging impaired response inhibition and salience attribution in human drug addiction: A systematic review. Neuron. (2018) 98:886–903. doi: 10.1016/j.neuron.2018.03.048 29879391 PMC5995133

[77] Weiland BJ Sabbineni A Calhoun VD Welsh RC Hutchison KE . Reduced executive and default network functional connectivity in cigarette smokers. Hum Brain Mapp. (2015) 36:872–82. doi: 10.1002/hbm.22672 PMC4978515 25346448

[78] Sridharan D Levitin DJ Menon V . A critical role for the right fronto-insular cortex in switching between central-executive and default-mode networks. Proc Natl Acad Sci U.S.A. (2008) 105:12569–74. doi: 10.1073/pnas.0800005105 PMC2527952 18723676

[79] Lerman C Gu H Loughead J Ruparel K Yang Y Stein EA . Large-scale brain network coupling predicts acute nicotine abstinence effects on craving and cognitive function. JAMA Psychiatry. (2014) 71:523–30. doi: 10.1001/jamapsychiatry.2013.4091 PMC4097018 24622915

[80] Hu W Fu X Qian R Wei X Ji X Niu C . Changes in the default mode network in the prefrontal lobe, posterior cingulated cortex and hippocampus of heroin users. Neural Regener Res. (2012) 7:1386–91. doi: 10.3969/j.issn.1673-5374.2012.18.004 PMC4308788 25657671

[81] Gorelick DA Zangen A George MS . Transcranial magnetic stimulation in the treatment of substance addiction. Ann N Y Acad Sci. (2014) 1327:79–93. doi: 10.1111/nyas.2014.1327.issue-1 25069523 PMC4206564

[82] Li X Hartwell KJ Owens M Lematty T Borckardt JJ Hanlon CA . Repetitive transcranial magnetic stimulation of the dorsolateral prefrontal cortex reduces nicotine cue craving. Biol Psychiatry. (2013) 73:714–20. doi: 10.1016/j.biopsych.2013.01.003 PMC3615051 23485014

[83] Tumati S Martens S de Jong BM Aleman A . Lateral parietal cortex in the generation of behavior: Implications for apathy. Prog Neurobiol. (2019) 175:20–34. doi: 10.1016/j.pneurobio.2018.12.003 30590096

[84] Raichle ME MacLeod AM Snyder AZ Powers WJ Gusnard DA Shulman GL . A default mode of brain function. Proc Natl Acad Sci U.S.A. (2001) 98:676–82. doi: 10.1073/pnas.98.2.676 PMC14647 11209064

[85] Whitfield-Gabrieli S Ford JM . Default mode network activity and connectivity in psychopathology. Annu Rev Clin Psychol. (2012) 8:49–76. doi: 10.1146/annurev-clinpsy-032511-143049 22224834

[86] Callard F Smallwood J Margulies DS . Default positions: how neuroscience’s historical legacy has hampered investigation of the resting mind. Front Psychol. (2012) 3:321. doi: 10.3389/fpsyg.2012.00321 22973252 PMC3437462

[87] Durazzo TC Meyerhoff DJ . GABA concentrations in the anterior cingulate and dorsolateral prefrontal cortices: Associations with chronic cigarette smoking, neurocognition, and decision making. Addict Biol. (2021) 26:e12948. doi: 10.1111/adb.12948 33860602 PMC8697713

[88] Brody AL Mandelkern MA Lee G Smith E Sadeghi M Saxena S . Attenuation of cue-induced cigarette craving and anterior cingulate cortex activation in bupropion-treated smokers: a preliminary study. Psychiatry Res. (2004) 130:269–81. doi: 10.1016/j.pscychresns.2003.12.006 PMC2773650 15135160

[89] Menon V Uddin LQ . Saliency, switching, attention and control: a network model of insula function. Brain Struct Funct. (2010) 214:655–67. doi: 10.1007/s00429-010-0262-0 PMC2899886 20512370

[90] Newberg A Lerman C Wintering N Ploessl K Mozley PD . Dopamine transporter binding in smokers and nonsmokers. Clin Nucl Med. (2007) 32:452–5. doi: 10.1097/01.rlu.0000262980.98342.dd 17515751

[91] Chen Y Fowler CH Papa VB Lepping RJ Brucks MG Fox AT . Adolescents’ behavioral and neural responses to e-cigarette advertising. Addict Biol. (2018) 23:761–71. doi: 10.1111/adb.2018.23.issue-2 PMC5636647 28401670

[92] Huang AS Mitchell JA Haber SN Alia-Klein N Goldstein RZ . The thalamus in drug addiction: from rodents to humans. Philos Trans R Soc Lond B Biol Sci. (2018) 373. doi: 10.1098/rstb.2017.0028 PMC5790826 29352027

[93] Roy DS Zhang Y Halassa MM Feng G . Thalamic subnetworks as units of function. Nat Neurosci. (2022) 25:140–53. doi: 10.1038/s41593-021-00996-1 PMC9400132 35102334

[94] Buckner RL . The cerebellum and cognitive function: 25 years of insight from anatomy and neuroimaging. Neuron. (2013) 80:807–15. doi: 10.1016/j.neuron.2013.10.044 24183029

[95] Moulton EA Elman I Becerra LR Goldstein RZ Borsook D . The cerebellum and addiction: insights gained from neuroimaging research. Addict Biol. (2014) 19:317–31. doi: 10.1111/adb.2014.19.issue-3 PMC4031616 24851284

[96] Martin-Sölch C Magyar S Künig G Missimer J Schultz W Leenders KL . Changes in brain activation associated with reward processing in smokers and nonsmokers. A positron emission tomography study. Exp Brain Res. (2001) 139:278–86. doi: 10.1007/s002210100751 11545466

[97] Paterson D Nordberg A . Neuronal nicotinic receptors in the human brain. Prog Neurobiol. (2000) 61:75–111. doi: 10.1016/S0301-0082(99)00045-3 10759066

