# Supplementary material for: The structural and functional brain alternations in tobacco use disorder: a systematic review and meta-analysis
Source: Front Psychiatry. 2025 Apr 11;16:1403604. doi: 10.3389/fpsyt.2025.1403604 (PMC12022757; doi:10.3389/fpsyt.2025.1403604)
Supplement: Supplementary Figure 1 — Funnel plots for significant results. [file DataSheet1.docx]

Supplementary Material

# Supplementary Figures and Tables

## Supplementary Figures


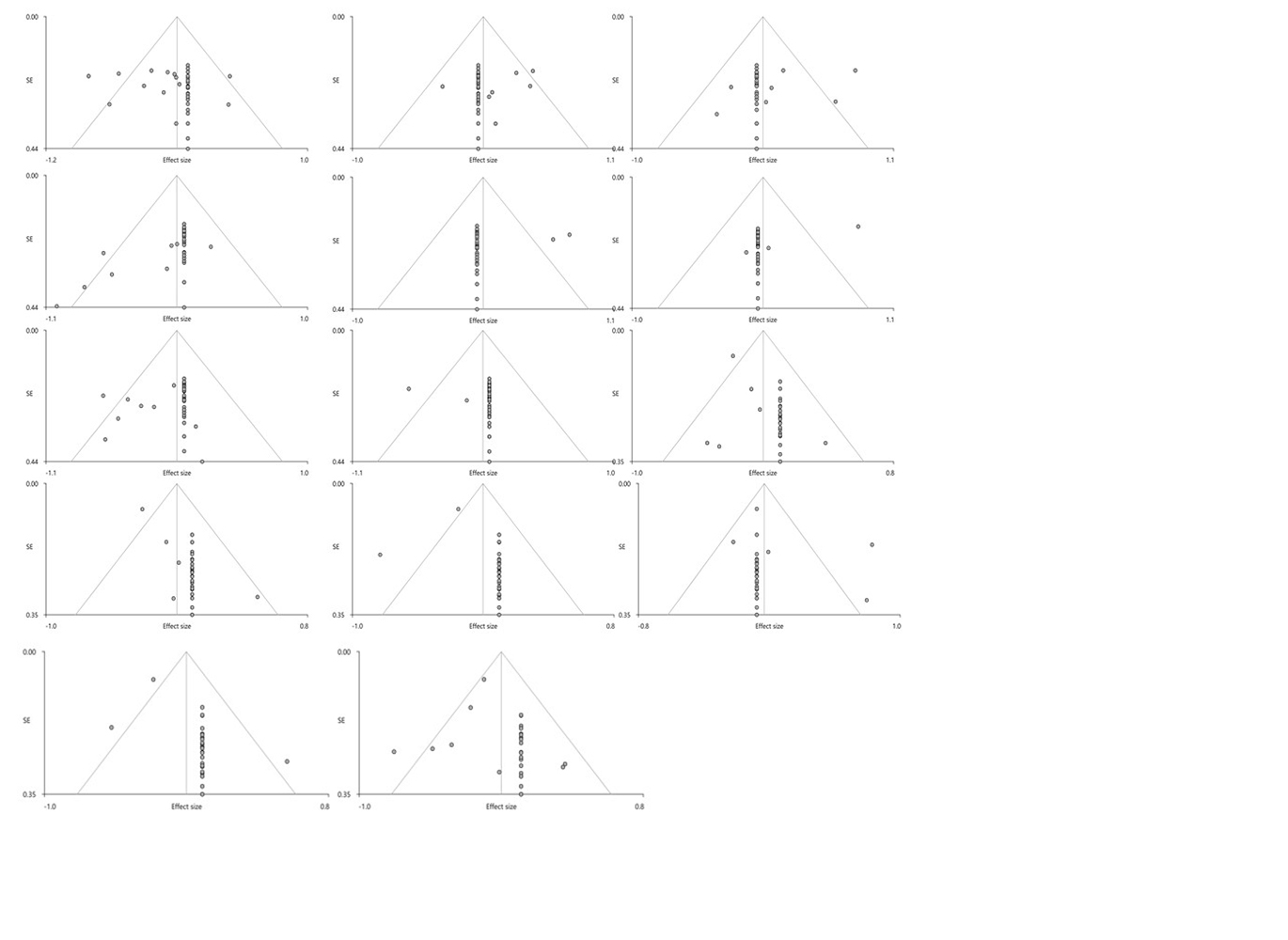


FIGURE S 1 Funnel plots for significant results.

## Supplementary Tables

TABLE S 1 Clinical characteristics and Technical details of rs-fMRI and VBM studies in TUD

| Study | Clinical characteristics | | | | Technical details | | | P-value |
| --- | --- | --- | --- | --- | --- | --- | --- | --- |
|  | Smoking history | Cigarette/day | Pack-years | FTND | MRI Scanner | Software | Coordinate System |  |
| 1. rs-fMRI studies | | | | | | | | |
| Akkermans et al. (2018) | 7.20(3.01) | 19.12(3.37) | NA | 3.80(1.83) | 3.0-T Siemens | SPM8 | MNI | P<0.05(FWE corrected) |
| Bi et al. (2017) | 4.20(1.88) | 15.58(5.53) | 3.46(2.40) | 5.73(2.02) | 3.0-T GE | FSL | MNI | P<0.05(FWE corrected) |
| Chen et al. (2014) | NA | NA | NA | 6.51(2.11) | 3.0-T GE | SPM8 | MNI | P<0.05(AlphaSim corrected) |
| Zhou et al. (2017) | 15.05(8.68) | 22.43(7.03) | 18.82(14.93) | 7.00(1.73) | 3.0-T Siemens | SPM12 | MNI | P<0.05(AlphaSim corrected) |
| Ge et al. (2017) | NA | NA | NA | 6.52(2.11) | 3.0-T GE | SPM8 | MNI | P<0.05(AlphaSim corrected) |
| Lin et al. (2021) | 5.90(2.81) | NA | NA | 6.40(2.20) | 3.0-T GE | FreeFurfer | MNI | P<0.05(FDR corrected) |
| Niu et al. (2023) | NA | NA | 21.64(9.72) | 4.91(1.93) | 3.0-T Siemens | SPM12 | MNI | P<0.005(GFR corrected) |
| Qiu et al. (2022) | NA | NA | NA | NA | 3.0-T Philips | SPM12 | MNI | P<0.05(GFR corrected) |
| Qiu et al. (2022) | NA | NA | NA | NA | 3.0-T Philips | SPM12 | MNI | P<0.05(GFR corrected) |
| Shen et al. (2017) | 17.36(6.58) | 23.46(9.53) | 20.63(12.28) | 5.18(2.18) | 3.0-T GE | SPM8 | MNI | P<0.05(FDR corrected) |
| Shen et al. (2018) | 17.35(6.61) | 23.56(9.55) | 20.70(12.33) | 5.23(2.14) | 3.0-T GE | SPM8 | MNI | P<0.05  (uncorrected) |
| Stoeckel et al. (2016) | 17.63(10.49) | 16.00(4.84) | 16.09(12.17) | 4.44(2.16) | 3.0-T Siemens | SPM8 | MNI | P<0.001(FWE corrected) |
| Tan et al. (2021a) | 32.1(11.49) | NA | NA | 5.45(2.04) | 3.0-T GE | SPM8 | MNI | P<0.01(AlphaSim corrected) |
| Wang, Bai, et al. (2017) | 4.70(1.50) | 16.6(5.30) | 3.90(1.80) | 6.10(1.30) | 3.0-T GE | SPM12 | MNI | P<0.05(FWE corrected) |
| Zhang, Zeng, et al. (2023) | NA | NA | NA | NA | 3.0-T Siemens | SPM12 | MNI | P<0.01(GFR corrected) |
| Yip et al. (2022) | 29.90(11.90) | NA | NA | 5.45(2.04) | 3.0-T Siemens | SPM8 | MNI | P<0.01  (uncorrected) |
| Qiu et al. (2021) | NA | NA | NA | NA | 3.0-T Philips | SPM8 | MNI | P<0.05(GFR corrected) |
| Qiu et al. (2021) | NA | NA | NA | NA | 3.0-T Philips | SPM8 | MNI | P<0.05(GFR corrected) |
| Wang et al. (2021) | 4.38(2.77) | 15.40(5.60) | 2.41(0.90) | 6.54(1.46) | 3.0-T Philips | FSL | MNI | P<0.05(FWE corrected) |
| Claus and Weywadt (2020) | 36.80(10.20) | NA | 35.8(21.4) | 5.20(2.50) | 3.0-T Siemens | FSL | MNI | P<0.05(FDR corrected) |
| Zhang et al. (2017) | 15.80(9.70) | 14.8(6.20) | NA | 3.90(2.50) | 3.0-T Siemens | SPM8 | MNI | P<0.05  (uncorrected) |
| Huang et al. (2014) | NA | NA | NA | 4.00(1.69) | 3.0-T Philips | SPM8 | MNI | P<0.001  (uncorrected) |
| Chen and Mo (2017) | 5.00(NA) | 10.00(NA) | 10.20(NA) | 5.10(1.50) | 3.0-T Siemens | SPM8 | MNI | P<0.005(AlphaSim corrected) |
| Yu et al. (2013) | 21.10(3.90) | 20.60(7.40) | 6.98(3.84) | 7.19(1.42) | 3.0-T Siemens | SPM8 | MNI | P<0.001  (uncorrected) |
| Wu et al. (2015) | 25.42(9.10) | 37.26(12.10) | 4.76(1.20) | 8.87(0.72) | 3.0-T Siemens | SPM8 | MNI | P<0.05(AlphaSim corrected) |
| Tang et al. (2012) | 10.20(5.80) | 20.30(7.60) | 6.32(4.47) | 6.41(1.70) | 3.0-T Siemens | SPM5 | MNI | P<0.01(AlphaSim corrected) |
| Wang, Qian, et al. (2017) | 18.97(6.60) | 23.28(9.89) | 22.03(12.07) | 5.16(2.00) | 3.0-T GE | SPM8 | MNI | P<0.05(AlphaSim corrected) |
| Wen et al. (2022) | 5.78(2.84) | NA | NA | 6.53(2.06) | 3.0-T GE | SPM12 | MNI | P<0.05(FDR corrected) |
| Zhang et al. (2020) | NA | NA | NA | NA | 3.0-T Philips | SPM8 | MNI | P<0.05(GFR corrected) |
| Liu et al. (2018) | 7.10(8.76) | 17.05(8.94) | 6.25(5.82) | 5.33(2.60) | 3.0-T GE | SPM8 | MNI | P<0.05(AlphaSim corrected) |
| Qiu et al. (2020) | 5.18(2.92) | NA | NA | 6.28(2.25) | 3.0-T GE | SPM8 | MNI | P<0.05(FWE corrected) |
| Niu et al. (2023) | NA | 4.54(3.97) | 21.64(9.72) | 4.91(1.93) | 3.0-T Philips | SPM12 | MNI | P<0.005(GFR corrected) |
| Gao et al. (2023) | 13.07(7.09) | 21.34(34.10) | 13.95(12.09) | 4.63(1.75) | 3.0-T Philips | SPM12 | MNI | P<0.005(GFR corrected) |
| Chu et al. (2014) | 20.00(8.00) | 12.00(5.00) | 8.33(12.00) | 5.00(3.00) | 3.0-T Philips | SPM8 | MNI | P<0.05(FDR corrected) |
| Wang, Shen, et al. (2017) | 19.00(6.10) | 23.5(10.2) | 4.43(4.12) | 5.20(2.10) | 3.0-T GE | SPM8 | MNI | P<0.005  (uncorrected) |
| Gao et al. (2023) | 13.07(7.09) | 6.64(2.89) | 13.95(12.09) | 4.63(1.75) | 3.0-T Siemens | SPM8 | MNI | P<0.005(GFR corrected) |
| Tan et al. (2021b) | 32.1(11.49) | NA | NA | 5.45(2.04) | 3.0-T GE | SPM8 | MNI | P<0.01(AlphaSim corrected) |
| 1. VBM studies | | | | | | | | |
| Conti (2022) | NA | 15.6(4.7) | 10.6(10.1) | 5.3(1.8) | 3.0-T Siemens | SPM12 | MNI | P<0.01(corrected) |
| Conti (2021) | NA | 15.0(4.5) | 10.4(8.1) | 5.0(1.5) | 3.0-T Siemens | SPM12 | MNI | P<0.01(corrected) |
| Zhang, Gao, et al. (2023) | 11.82(5.77) | 16.11(8.35) | 10.08(7.95) | 3.45(2.03) | 3.0-T GE | SPM12 | MNI | P<0.001(GRF corrected) |
| Daniju et al. (2022) | 6.2(4.2) | 6.6(5.3) | 2.7(3.65) | NA | 3.0-T Siemens | SPM12 | MNI | P<0.05(FWE corrected) |
| Kunas et al. (2020) | NA | NA | NA | NA | 3.0-T Philips | SPM12 | MNI | P<0.05(FWE corrected) |
| Ye et al. (2020) | 25.34(9.23) | 35.13(10.70) | NA | 8.89(0.68) | 3.0-T Siemens | SPM8 | MNI | P<0.05(FWE corrected) |
| Chen et al. (2022) | 15.14(3.34) | NA | NA | 4.83(0.88) | 3.0-T Siemens | CAT12 | MNI | P<0.05(FWE corrected) |
| Cai et al. (2022) | 23.8(7.9) | 0.26(0.2) | NA | 8.89(0.71) | 3.0-T Siemens | SPM8 | MNI | P<0.05(AlphaSim corrected) |
| Bu et al. (2016) | 4.27(2.44) | 15.04(4.82) | 3.55(2.97) | 4.42(2.20) | 3.0-T Philips | SPM8 | MNI | P<0.05(FWE corrected) |
| Franklin et al. (2014) | 16.0(11.3) | 16.1(6.1) | 13.9(9.9) | 4.6(1.7) | 3.0-T Siemens | SPM8 | MNI | P<0.025(FWE corrected) |
| Hanlon et al. (2016) | 15(NA) | 15.3(6.5) | 5.2(0.4) | 4.3(2.3) | 3.0-T Siemens | SPM8 | MNI | P<0.01(FWE corrected) |
| Fritz et al. (2014) | 26.8(NA) | 13.17(6.99) | 17.81(12.25) | NA | 1.5-T Siemens | SPM8 | MNI | P<0.05(FWE corrected) |
| Gallinat et al. (2006) | 13.9(7.3) | 14.5(9.2) | 13.5(13.0) | 2.9(1.7) | 3.0-T Siemens | SPM2 | MNI | P<0.05(FDR corrected) |
| Morales et al. (2012) | 29.6(0.3) | 14.1(1.2) | 11.5(1.9) | 3.8(0.4) | 1.5-T Siemens | SPM8 | MNI | P<0.05(FWE corrected) |
| Peng et al. (2018) | NA | 38.70(8.36) | 31.06(7.40) | NA | 3.0-T Siemens | SPM8 | MNI | P<0.05(AlphaSim corrected) |
| Peng et al. (2018) | NA | 16.15(5.16) | 8.77(3.57) | NA | 3.0-T Siemens | SPM8 | MNI | P<0.05(AlphaSim corrected) |
| Qian et al. (2019) | 18.9(6.4) | 23.6(10.4) | NA | 5.4(2.4) | 3.0-T GE | SPM8 | MNI | P<0.05(GRF corrected) |
| Stoeckel et al. (2016) | 17.6(10.49) | 16.0(12.17) | 16.09(12.2) | 4.44(2.16) | 3.0-T Siemens | SPM8 | MNI | P<0.05(FWE corrected) |
| Wang et al. (2014) | 4.95(2.27) | 11.9(6.13) | 3.1(2.63) | NA | 3.0-T Siemens | SPM8 | MNI | P<0.05(AlphaSim corrected) |
| Peng et al. (2017) | NA | 34.97(7.31) | 23.34(13.33) | 8.0(1.02) | 3.0-T Siemens | SPM8 | MNI | P<0.05(AlphaSim corrected) |
| Peng et al. (2017) | NA | 18.07(6.99) | 15.9(10.57) | 3.43(1.65) | 3.0-T Siemens | SPM8 | MNI | P<0.05(AlphaSim corrected) |
| Yu (2011) | 21.1(3.9) | 20.6(7.4) | NA | 7.19(1.42) | 3.0-T Siemens | SPM5 | MNI | P<0.05(FWE corrected) |
| Zhang et al. (2011) | 12.8(7.4) | 20.9(6.6) | 12.9(7.9) | 5.4(1.9) | 3.0-T Siemens | FSL | MNI | P<0.01(FWE corrected) |
| Zorlu et al. (2017) | 12.4(7.1) | NA | NA | 5.8(2.2) | 1.5-T Philips | FreeSurfer | MNI | P<0.05(uncorrected) |
| Shen et al. (2018) | 17.36(6.58) | 23.46(9.53) | 20.63(12.28) | 5.18(2.18) | 3.0-T GE | SPM8 | MNI | P<0.05(FDR corrected) |
| Liao et al. (2012) | 10.4(5.72) | 20.3(7.7) | NA | NA | 3.0-T Siemens | SPM5 | MNI | P<0.001(uncorrected) |
| Brody et al. (2004) | NA | 26.2(7.4) | 31.0(17.9) | 5.1(1.9) | 1.5-T Siemens | SPM99 | MNI | P<0.001(uncorrected) |
| Faulkner et al. (2021) | NA | 11.45(4.73) | 6.21(5.37) | NA | 3.0-T Siemens | SPM12 | MNI | P<0.05(FWE corrected) |

Abbreviations: TUD, tobacco use disorder; HCs, healthy controls; ReHo, regional homogeneity; ALFF, amplitude of low-frequency fluctuations; fALFF, fractional amplitude of low-frequency fluctuations; FC, functional connectivity; NA, not available; DSM, The diagnostic and statistical manual of mental disorders.

TABLE S 2 Brain locations of FC, ReHo, ALFF, and fALFF differences in individuals with TUD compared to HCs

| Methods | Anatomical region | MNI coordinate | Number of voxels | SDM-z | P value | BA |
| --- | --- | --- | --- | --- | --- | --- |
| FC | TUD>HC |  |  |  |  |  |
|  | Left inferior parietal gyrus | -40,-58,52 | 194 | 1.465 | 0.000541866 | 40 |
|  | Left supplementary motor area | -6,16,62 | 38 | 1.227 | 0.001899183 | 6 |
|  | TUD<HC |  |  |  |  |  |
|  | Right caudate nucleus | 16,22,6 | 154 | -1.739 | 0.000608981 | NA |
|  | Left inferior frontal gyrus, triangular part | -52,28,2 | 152 | -1.557 | 0.002177835 | 45 |
|  | Right cuneus cortex | 4,78,30 | 61 | -1.615 | 0.001470804 | 18 |
| ReHo | TUD>HC |  |  |  |  |  |
|  | Right cerebellum, crus II | 36,-84,-34 | 428 | 1.455 | 0.002368808 | NA |
|  | Left superior parietal gyrus | -16,68,54 | 151 | 1.675 | 0.000918627 | 7 |
|  | TUD<HC |  |  |  |  |  |
|  | Right middle frontal gyrus | 46,44,2 | 1123 | -2.404 | 0.000010312 | 45 |
|  | Right superior frontal gyrus, dorsolateral | 16,52,32 | 304 | -1.837 | 0.000443816 | 9 |
|  | Left cerebellum, crus I | -44,-56,-34 | 117 | -1.455 | 0.003168762 | NA |
| ALFF | TUD>HC |  |  |  |  |  |
|  | Left superior frontal gyrus, dorsolateral | -20,64,22 | 510 | 1.889 | 0.000149667 | 10 |
|  | Left middle frontal gyrus | -28,38,26 | 264 | 1.414 | 0.000949562 | 46 |
|  | Corpus callosum | -10,50,36 | 203 | 1.413 | 0.000949562 | NA |
|  | TUD<HC |  |  |  |  |  |
|  | Left cerebellum, hemispheric lobule IV / V | -16,-38,-20 | 568 | -1.151 | 0.001826942 | 30 |
| fALFF | TUD>HC |  |  |  |  |  |
|  | Right caudate nucleus | 8,14,6 | 263 | 1.424 | 0.000309646 | 25 |
|  | Left calcarine fissure | -6,16,62 | 240 | 1.039 | 0.002544284 | 17 |
|  | Right median network, cingulum | 18,-36,-2 | 133 | 1.015 | 0.002895236 | NA |
|  | Left fusiform gyrus | -38,-66,-16 | 82 | 1.009 | 0.003050029 | 19 |
|  | Left inferior network, inferior longitudinal  fasciculus | -22,-78,-4 | 29 | 1.009 | 0.003081024 | NA |
|  | TUD<HC |  |  |  |  |  |
|  | Left precuneus | 0,-52,52 | 574 | -1.424 | 0.000299335 | NA |
|  | Right inferior temporal gyrus | 48,-60,-16 | 353 | -1.420 | 0.000356078 | 37 |

TABLE S 3 Subgroup analysis results for VBM and rs-fMRI studies in TUD (adults group and adolescents group)

| Age | Anatomical region | MNI coordinate | Number of voxels | SDM-z | P value | BA |
| --- | --- | --- | --- | --- | --- | --- |
| VBM results | | | | | | |
| Adults | TUD>HC |  |  |  |  |  |
|  | Right lingual gyrus | 18,-46,0 | 339 | 1.255 | 0.000061929 | 27 |
|  | TUD<HC |  |  |  |  |  |
|  | Left insula | -38,-20,0 | 1201 | -3.797 | 0.000005186 | 48 |
|  | Right superior frontal gyrus, medial orbital | 6,24,-14 | 195 | -3.283 | 0.000185788 | 11 |
|  | Right anterior cingulate/ paracingulate gyri | 8,24,2 | 89 | -2.956 | 0.000810266 | 10 |
|  | Left superior frontal gyrus, orbital part | -8,62,-16 | 67 | -3.086 | 0.000423193 | 11 |
|  | Right middle frontal gyrus, orbital part | 38,52,-2 | 34 | -2.774 | 0.001589537 | 46 |
| Adolescents | TUD>HC |  |  |  |  |  |
|  | Left striatum | -20,2,2 | 2217 | 2.157 | ~0 | NA |
|  | Right supramarginal gyrus | 60,-48,34 | 116 | 1.033 | 0.002167523 | 40 |
|  | TUD<HC |  |  |  |  |  |
|  | Left anterior cingulate/ paracingulate gyri | -2,36,22 | 1137 | -1.605 | 0.000118673 | 32 |
|  | Right lenticular nucleus | 34,14,-2 | 211 | -1.011 | 0.002203643 | 48 |
|  | Left anterior thalamic projections | -36,14,-2 | 157 | -1.009 | 0.002296567 | NA |
|  | Right thalamus | 6,-10,10 | 73 | -1.012 | 0.002162397 | NA |
| rs-fMRI results | | | | | | |
| Adults | TUD>HC |  |  |  |  |  |
|  | Left cerebellum, hemispheric lobule IV / V | -18,-52,-22 | 266 | 1.221 | 0.001486301 | 37 |
|  | Left superior frontal gyrus, medial | -4,24,38 | 209 | 1.185 | 0.001692772 | 32 |
|  | Left inferior parietal gyri | -42,-54,56 | 199 | 1.310 | 0.000928938 | 40 |
|  | Left superior frontal gyrus, dorsolateral | -18,68,16 | 89 | 1.408 | 0.000614166 | 10 |
|  | TUD<HC |  |  |  |  |  |
|  | Right superior frontal, medial orbital | 8,42,-10 | 2673 | -2.949 | ~0 | 11 |
|  | Left precuneus | -2,-56,36 | 958 | -1.710 | 0.000423193 | NA |
|  | Right middle frontal gyrus | 46,44,8 | 33 | -1.472 | 0.001568913 | 45 |
| Adolescents | TUD>HC |  |  |  |  |  |
|  | Right superior frontal gyrus, medial | 2,62,-4 | 453 | 1.611 | 0.001026988 | 10 |
|  | Left median network, cingulum | -6,-70,36 | 164 | 1.506 | 0.001522422 | NA |
|  | Left supplementary motor area | -12,20,64 | 21 | 1.307 | 0.003896415 | 8 |
|  | TUD<HC |  |  |  |  |  |
|  | Left anterior cingulate/ paracingulate gyri | -6,30,30 | 423 | -1.565 | 0.001754701 | 32 |
|  | Left anterior thalamic projections | -16,12,14 | 262 | -1.571 | 0.001687586 | NA |
|  | Right caudate nucleus | 14,22,8 | 13 | -1.510 | 0.002404928 | NA |
